# LncRNA HAGLROS promotes breast cancer evolution through miR-135b-3p/COL10A1 axis and exosome-mediated macrophage M2 polarization

**DOI:** 10.1038/s41419-024-07020-x

**Published:** 2024-08-28

**Authors:** Ziqi Meng, Rui Zhang, Xuwei Wu, Zhengri Piao, Meihua Zhang, Tiefeng Jin

**Affiliations:** 1 Department of Central Laboratory, Yanbian University Hospital, Yanji, China; 2 Department of Pathology and Cancer Research Center, Yanbian University, Yanji, China; 3 Department of Pathology, Chifeng Municipal Hospital, Chifeng, China; 4 Department of Radiology, Yanbian University Hospital, Yanji, Jilin China; 5 Department of Health Examination Centre, Yanbian University Hospital, Yanji, China

**Keywords:** Cell invasion, Breast cancer, Transcriptomics, Long non-coding RNAs

## Abstract

Long non-coding RNAs (lncRNAs) play an important role in breast cancer progression, but the function of lncRNAs in regulating tumor-associated macrophages (TAMs) remains unclear. As carriers of lncRNAs, exosomes play an important role as mediators in the communication between cancer cells and the tumor microenvironment. In this study, we found that lncRNA HAGLROS was highly expressed in breast cancer tissues and plasma exosomes, and its high expression was related to the poor prognosis of breast cancer patients. Functionally, breast cancer cell-derived exosomal lncRNA HAGLROS promotes breast cancer cell proliferation, migration, epithelial-mesenchymal transition (EMT) process and angiogenesis by inducing TAM/M2 polarization. Mechanistically, lncRNA HAGLROS competitively binds to miR-135-3p to prevent the degradation of its target gene COL10A1. Collectively, these results indicated that the lncRNA HAGLROS/miR-135b-3p/COL10A1 axis promoted breast cancer progression, and revealed the interactive communication mechanism between breast cancer cells and TAMs, suggesting that lncRNA HAGLROS may be a potential biomarker and therapeutic target for breast cancer.

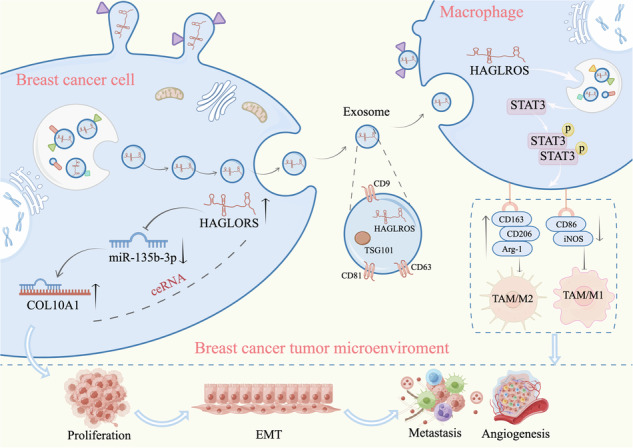

## Background

Breast cancer is the most commonly diagnosed cancer and the leading cause of cancer deaths in women. According to the latest global cancer statistics, the incidence of breast cancer in women has overtaken lung cancer as the leading cause of cancer incidence worldwide in 2020. Among women, breast cancer ranks first in terms of incidence and mortality and is a serious health risk for women [1, 2]. Despite advances in early diagnosis and treatment of different subtypes of breast cancer, the prognosis of patients with advanced and metastatic breast cancer remains poor. Therefore, searching for effective biomarkers is clinically important for the treatment of breast cancer patients.

Less than 2% of the human genome is transcribed as coding RNA, while more than 98% is transcribed as non-coding RNA (ncRNA), indicating that a large amount of ncRNAs is stored in the human genome [3]. LncRNA is ncRNA that is more than 200 nucleotides in length and does not have the potential to encode proteins due to the lack of effective open reading frame (ORF) [4]. Several studies have found that aberrantly expressed lncRNA is closely associated with the development of many types of tumors [5–7]. miRNA is a group of endogenous non-coding single-stranded small RNAs, ~18–22 nucleotides in length and widely found in eukaryotes, which bind fully or partially to the 3′-Untransated Regions (3′-UTR) of mRNAs by base complementary pairing to promote degradation or translational repression of target mRNAs at the post-transcriptional level [8]. Studies have reported that lncRNA affects tumor progression through multiple mechanisms, and lncRNA as competitive endogenous RNA (ceRNA) competitively binds to miRNA to affect mRNA expression is a hot topic of current research [9, 10]. For example, lncRNA CRART16 promotes gastric cancer angiogenesis by sponging miR-122-5p to upregulate FOS/VEGFD expression, and is a prognostic marker and therapeutic target for gastric cancer [11]. LncRNA HCG18 promotes proliferation, migration and EMT process in epithelial ovarian cancer by sponging miR-29a/b to upregulate TRAF4/TRAF5 expression [12]. However, the functions and mechanisms of most aberrantly expressed lncRNA as ceRNA in breast cancer remain unclear.

LncRNA HAGLROS (NR_110457.1), an antisense lncRNA of HOXD antisense growth-associated long-stranded noncoding RNA, which has been reported to be upregulated as an oncogene in a variety of tumors including nephroblastoma and laryngeal squamous cell carcinoma, and to play an important role in the process of tumor proliferation, metastasis, autophagy and apoptosis [13, 14]. Studies have reported that lncRNA HAGLROS act as ceRNA to regulate miRNA, thereby inhibiting the translation or degradation of target genes and promoting tumor progression [15, 16]. However, the molecular sponge of lncRNA HAGLROS as which miRNA regulates the biological behavior of breast cancer and its related mechanisms has not been reported.

Macrophages in the tumor microenvironment (TME), called TAMs, are one of the important immune cells in the TME and can be divided into two types: classically activated macrophage (TAM/M1) and alternatively activated macrophage (TAM/M2) [17]. TAM/M1 has pro-inflammatory and anti-tumor properties, while TAM/M2 has anti-inflammatory and pro-tumorigenic properties [18]. Due to the plasticity of TAMs, TAM/M1 and TAM/M2 states are not invariant and the two phenotypes can be interconverted under certain conditions, being dynamic and reversible [17]. Recruitment, polarization, and phenotypic transformation of TAMs regulate tumorigenesis development by inducing cancer cells proliferation, invasion, metastasis, drug resistance and immune escape [19]. Recent studies have shown that lncRNA plays an important role in the polarization process of TAMs and that they serve as diagnostic and therapeutic biomarkers. For example, lncRNA PCAT5 regulates the miR-326/KLF1 axis by inhibiting TAM/M2 polarization, thereby affecting the metastatic and EMT processes in non-small cell lung cancer cells [20].

Exosomes are extracellular vesicles with a 40–150 nm diameter, secreted by different cell types in the TME, which mediate intercellular interactions and regulate various pathophysiological processes by packaging proteins and nucleic acids [21]. Studies have reported that exosomes mediate the interaction between tumor cells and TAMs, and that exosome-packaged lncRNA is involved in a variety of physiological processes [22]. Tumor-derived exosomes (TEXs) can transport lncRNA to TAMs, which mediate TAMs polarization through signaling pathway activation, signal transduction, transcriptional and post-transcriptional regulation, and subsequently promote the malignant evolution of tumor cells [23]. In tumors, aberrantly expressed lncRNA, TAMs and exosomes can be used not only as diagnostic and prognostic markers, but also as potential targets for cancer therapy. However, whether TEXs regulate the polarization of TAMs by transferring lncRNA HAGLROS has not been reported yet and needs to be further explored.

Therefore, this study aimed to investigate the mechanism of lncRNA HAGLROS as ceRNA to regulate the malignant progression of breast cancer, reveal the regulatory mechanism of exosomal lncRNA HAGLROS between TAMs and breast cancer cells, and provide a potential target for breast cancer treatment.

## Material and methods

### Clinical tissue specimens

Breast cancer tissue microarrays were constructed by Shanghai Zhuoli Biotechnology Co., Ltd (Zhuoli Biotechnology Co, China). The microarrays contained 178 tissue specimens, including 99 breast cancer and 79 adjacent tissue specimens. The clinicopathological parameters of the 178 specimens included gender, age, pathological grade, tumor size, clinical stage, lymph node metastasis, distant metastasis, ER, PR, HER2 and Ki67 levels (Incomplete data on clinicopathological parameters in some cases).

### Human plasma samples

Plasma exosome samples from ten human breast cancer and healthy individuals were used to analyze the RNA levels of lncRNA HAGLROS, which were obtained from the Affiliated Hospital of Yanbian University, China. All patients did not receive chemotherapy or radiotherapy before surgery, and exosomes were extracted from plasma immediately after extraction and stored in a −80 °C refrigerator until further use.

### Cell culture

Human normal mammary epithelial cell line MCF-10A, breast cancer cell lines (MDA-MB-231, Hs578T, MDA-MB-468, MDA-MB-453, MCF-7, and SK-BR3), immortalized HUVECs, HLECs and human monocyte THP-1 cell line were purchased from the American Type Tissue Culture Collection (ATCC). All cell lines were tested and authenticated by their manufacturers. Cells were cultured in RPMI-DMEM (Gibco, USA) or 1640, which contained 10% FBS (Gibco, USA) and 1% streptomycin-penicillin (100 U/mL). THP-1 cells were differentiated into macrophages using 100 ng/mL phorbol-12-myristate 13-acetate (PMA; Sigma, USA) for 24 h. Cells were incubated at 37 °C in an atmosphere of 5% CO_2_.

### Plasmid construction and transfection

For stable transfection, the pmirGLO plasmid was used as a negative control, human Lenti-lncRNA HAGLROS-GFP, Lenti-sh lncRNA HAGLROS-GFP and corresponding empty vector were transfected into breast cancer cells. Following lentiviral transfection, puromycin was used to establish stable-expression cell lines. For transient transfection, COL10A1 overexpression plasmid, COL10A1 siRNA (control siRNA, si-COL10A1#1, si-COL10A1#2 and si-COL10A1#3), miR-135b-3p mimics and inhibitors were purchased from Beijing Syngenbio Co., LTD. Breast cancer cells were transfected with siRNA or miRNA and Lipofectamine 3000 (Invitrogen, USA) according to the manufacturer’s instructions. The sequences of siRNAs were listed in Supplementary Table 1.

### In situ hybridization (ISH)

Tumor tissue sections were dewaxed and dehydrated. Subsequently, antigen retrieval was performed using microwave heating in 10 mM citrate buffer (pH = 7.0) at 80 °C for 10 min. Tumor tissue sections were incubated overnight at 37 °C using digoxigenin-conjugated lncRNA HAGLROS probes (Hippo Bio, China). The ISH kit (BOSTER, China) was used to evaluate the expression of lncRNA HAGLROS. The sections were stained using DAB (ZSGB-BIO, China) at room temperature for 2 min and counterstained with hematoxylin. Subsequently, the images of ISH were captured using a microscope (Olympus IX73, Japan).

Two pathologists without knowledge of the clinical data scored all the tissue specimens. If there are differences, the final score is reassessed under a double-headed microscope. The staining intensity was graded 4 stages: “0” (negative), “1” (weak), “2” (moderate) and “3” (strong). The area of cancer cell expression was graded 5 stages: “0” (<5%), “1” (5%-25%), “2” (26%-50%), “3” (51%-75%) and “4” (>75%)。ISH score was a combination of positive intensity and cancer cell area. Briefly, IHC staining was scored as “0” (negative, −), “1–3” (weak, +), “4–6” (moderate, ++) and “8–12” (strong, +++).

### qRT-PCR assay

The total RNA was extracted with TRIzol reagent (Invitrogen, USA) according to the manufacturer’s instructions. For mRNA and lncRNA, reverse transcription was carried out using Reverse Transcription System (Promega, USA). qRT-PCR was performed using UltraSYBR Mixture (CWBio, China). For miRNA, reverse transcription and qRT-PCR were performed using the miDETECT A Track miRNA qRT-PCR Kit (RIBOBIO, China). GAPDH was used as an internal control for mRNA and lncRNA. U6 was used as an internal control for miRNA. Relative RNA abundances were calculated by the standard 2^−ΔΔCt^ method. The primers were listed in Supplementary Table 2.

### MTT assay

Cells (5 × 10^3^)/well were seeded and incubated in 96-well plates at 37 °C for 0, 24, 48, and 72 h. Subsequently, 100 μL MTT reagent (1 mg/mL) was added to each well and the cells were incubated at 37 °C for 4 h under the same conditions. Then, 100 μL DMSO was added to each well and the optical density at 490 nm was assessed. At least five wells/groups were analyzed and the experiment was repeated three times.

### Colony formation assay

Cells (5 × 10^2^)/well were seeded and incubated in six-well plates at 37 °C for 14 days. Subsequently, cells were fixed with 4% paraformaldehyde for 30 min and stained with crystal violet for 30 min. Statistical significance was calculated from each three independent experiments.

### EdU incorporation assay

Cells (5 × 10^4^)/well were seeded and incubated in 96-well plates at 37 °C for 48 h. The EdU incorporation kit (RIBOBIO, China) was used to evaluate cell proliferation. A fluorescence microscope (Olympus IX73, Japan) was used to obtain images.

### Animal studies

A total of 60 female BALB/c mice (age,4–5 weeks) were purchased from the Vital Rivers (Beijing, China) and randomly distributed into twelve groups. All mice were housed under specific pathogen-free conditions. Mice were used following the guidelines of the Yanbian University Animal Ethics Committee. The ethics committee allowed a maximum tumor size of 2000mm^3^.

For the xenograft model, each group of five and grouped as follows: (1) MDA-MB-231-Vector (5 × 10^6^ cells), MDA-MB-231-LncRNA HAGLROS (5 × 10^6^ cells); (2) Hs578T-Control (5 × 10^6^ cells), Hs578T-sh-LncRNA HAGLROS (5 × 10^6^ cells); 3) MDA-MB-231 (5 × 10^6^ cells), MDA-MB-231 (5 × 10^6^ cells)+THP-1 (5 × 10^5^ cells), MDA-MB-231 (5 × 10^6^ cells)+THP-1 (10 μg/mL EXO^231-vector^ pretreatment, 5 × 10^5^ cells), MDA-MB-231 (5 × 10^6^ cells)+THP-1 (10 μg/mL EXO^231-HAGLROS^ pretreatment, 5 × 10^5^ cells). The cells were injected subcutaneously into mice to establish a tumor model. Tumor size was measured every 3 days and tumor volume was calculated using the following formula: length × width^2^ × 0.5. The mice were sacrificed after 5 weeks.

For lung metastasis models, each group of five and grouped as follows: (1) MDA-MB-231-Vector (1 × 10^6^ cells), MDA-MB-231-LncRNA HAGLROS (1 × 10^6^ cells); (2) Hs578T-Control (1 × 10^6^ cells), Hs578T-sh-LncRNA HAGLROS (1 × 10^6^ cells). The cells were injected into the tail vein of the mice and then were sacrificed after 7 weeks. The lungs were collected and the fluorescence intensity was detected by UVP iBOX^®^ Scientia^TM^ 900 (Analytik Jena US, USA). Subsequently, the lungs were stained with Bouin’s solution and surface nodules were quantified. The tumor and lung tissues were fixed with 10% formalin at 4 °C for 24 h and the paraffin-embedded tumor tissues were sliced into 4-µm-thick sections. The expression of the markers was confirmed by immunohistochemical staining. Animal euthanasia was performed via cervical dislocation under 2% isoflurane anesthesia.

### Immunohistochemistry (IHC)

Tumor tissue sections were dewaxed and dehydrated. Subsequently, antigen retrieval was performed using microwave heating in 10 mM citrate buffer (pH = 7.0) at 80 °C for 20 min. Endogenous peroxidase was blocked with 3% H_2_O_2_ (ZSGB-BIO, China) at room temperature for 30 min. The tissue sections were incubated with primary antibody at 4 °C overnight. Following the primary incubation, the samples were incubated with horseradish peroxidase-conjugated secondary antibody (ZSGB-BIO, China) at room temperature for 1 h. The sections were stained using DAB (ZSGB-BIO, China) at room temperature for 5 min and counterstained with hematoxylin. Subsequently, the images of IHC were captured using a microscope (Olympus IX73, Japan).

### Wound healing assay

Cells with 80–90% fusion rate were incubated in six-well plates at 37 °C for 24 h. The wound was scratched vertically using a 200 μL pipette tip and washed three times with PBS to remove dead cells in the well. The cells with serum-free culture were imaged (Olympus IX73, Japan) at 0, 24, and 48 h. Wound width was measured by Image J software and then analyzed by the cell migration rate calculation formula. Cell Migration Rate= (Initial wound Width-Wound Width after 24/48 h)/Initial Wound Width × 100%.

### Transwell assay (migration and invasion)

Invasion experiments were performed in advance by diluting Matrigel (BD Biosciences, USA) and RPMI-DMEM at a 1:1 ratio in 24-well plates and solidified at 37 °C for 4 h. The subsequent steps are the same as for the Migration experiment. Cells (5 × 10^4^) in 100 μL serum-free RPMI-DMEM were seeded into the upper chamber. The bottom chamber was filled with 800 μL of 10% FBS in RPMI-DMEM. The cells were incubated at 37 °C (culture time was developed according to the cells). Cells passing through the subsurface of the filtration membrane were fixed with 4% paraformaldehyde at room temperature for 20 min and were then stained with 0.1% hematoxylin at room temperature for 20 min. Three fields (magnification, × 200) were randomly selected for imaging using a microscope (Olympus IX73, Japan). Image-J software (v. 1.46; National Institutes of Health) was used to quantify the number of cells in each field.

### Western blotting

Cells were collected and the total protein was extracted using RIPA lysate (RIPA lysis buffer: PMSF = 100:1). The protein concentration was determined and quantified using a BCA kit (CWBio, China). Proteins were separated using SDS-PAGE and separated proteins were then transferred to the PVDF membrane (Millipore, Sigma). The membrane was placed in 5% non-fat milk (BD Biosciences, USA) and blocked for 2 h to remove nonspecific binding sites. The membranes were incubated with the primary antibody at 4 °C overnight and then the secondary antibody at room temperature for 1 h. An enhanced chemiluminescence kit (ZOMANBIO, China) was used to detect antibody signals images were collected. The antibodies are listed in Supplementary Table 3.

### Immunofluorescence (IF) staining

Cells with 30–50% fusion rate were seeded into six-well plates at 37 °C for 48 h. The cells were fixed with anhydrous methanol for 15 min and permeated with 0.5% Triton X-100 (CWBio, China) at room temperature for 10 min. The cells were then blocked with 3% BSA (Solarbio, China) at room temperature for 2 h. Subsequently, cells were incubated with the primary antibody in 3% BSA at 4 °C overnight. Following the primary incubation, the cells were then incubated with Alexa Fluor 488 goat anti-rabbit IgG and Alexa Fluor 568 goat anti-mouse IgG at room temperature for 1 h. Cells were counterstained with DAPI and imaged by Leica SP5II confocal microscope (Leica Microsystems GmbH, Germany).

### Endothelial tube formation assay

Matrigel and RPMI-DMEM were diluted 1:1 in 96-well plates and solidified at 37 °C for 4 h. HUVECs or HLECs (3 × 10^4^) were incubated in 2:1 diluted CM and culture medium at 37 °C for 4 h. The capillary structure was imaged using a microscope (Olympus IX73, Japan).

### Vasculogenic mimicry (VM) assay

Matrigel and RPMI-DMEM were diluted 1:1 in 96-well plates and solidified at 37 °C for 4 h. Breast cancer cells (3 × 10^4^) were incubated in a culture medium at 37 °C for 4 h. The capillary structure was imaged using a microscope (Olympus IX73, Japan).

### Chic chorioallantoic membrane (CAM) assay

Fertilized eggs were incubated at a temperature of 37.5 ± 0.5 °C and about 70% humidity for 5–7 days. Then, a small window was cut in the eggshell to expose the CAM, and a silicone ring was placed on the allantoic membrane without large blood vessels and 3 × 10^6^ pretreated cells were injected into the ring. Next, the window is covered with tape and incubated for 48 h. The CAM was observed and images were captured under a stereomicroscope (Olympus SZX10, Japan). All experiments were performed in accordance with the procedures of the Animal Ethics Committee of Yanbian University.

### Fluorescence in situ hybridization (FISH)

Cells with 50% fusion rate were seeded into six-well plates at 37 °C for 48 h. The cells were fixed with 4% paraformaldehyde for 10 min and permeated with 0.5% Triton X-100 at 4 °C for 10 min. Then, cells were hybridized using the FISH kit (RIBOBIO, China) according to the manufacturer’s instructions. Cells were incubated overnight at 37 °C using fluorescence-conjugated lncRNA HAGLROS probes (RIBOBIO, China). Subsequently, cells were counterstained with DAPI and imaged by Leica SP5II confocal microscope (Leica Microsystems GmbH, Germany).

### RNA sequencing

RNA extraction and sample preparation were performed on Hs578T-control and Hs578T-sh-lnRNA HAGLROS cells. The total RNA was extracted with TRIzol reagent, followed by digestion with Dnase I to remove residual DNA. The samples were sent to a gene sequencing company (APTBIO, China) for transcriptome sequencing to detect the level of miRNA expression regulated by lncRNA HAGLROS. For transfection cells, *P* value was less than 0.05 and the log_2_ | FC| was greater than 1. Heat map and volcanic map were generated by R software and significance was determined using Student’s *t* test.

### Dual luciferase reporter assay

The wild-type or mutant lncRNA HAGLROS or 3′UTR of COL10A1 was amplified and cloned into pmirGLO vector, respectively. Then, cells were incubated in 96-well plates, and Lipofectamine 3000 was cotransfected with wild-type or mutant luciferase plasmids and miR-135b-3p or control miRNA. Firefly and Renilla luciferase expressions were measured post-transfection using the Dual-Luciferase Kit (Promega, USA) according to the manufacturer’s instructions.

### RNA immunoprecipitation (RIP) assay

A RIP RNA-Binding Protein Immunoprecipitation Kit (GENESEED, China) was used to determine the relationship between COL10A1 and lncRNA HAGLROS and miR-135b-3p. Antibodies used for the RIP assay included anti-COL10A1 and control IgG (Abcam, USA). Coprecipitated RNA was used for cDNA synthesis and evaluated by qRT-PCR.

### Exosome extraction and identification

Cells were seeded and incubated in a 10 cm dish at 37 °C for 48 h. Subsequently, discard the supernatant and add 10 mL of serum-free medium for 48 h. Collect supernatant, centrifuge at 4 °C for 15 min, transfer supernatant to a new EP tube and add 2 mL of ExoQuick reagent (SBI, USA) overnight at 4 °C. Centrifuge at 4 °C for 30 min and carefully discard the supernatant. Exosomes were suspended in 100–500 μL RNase free water and stored at −80 °C. TEM, NTA and western blotting were used to identify the exosomes.

### Exosome uptake assay

PKH26, a red fluorescent dye (Sigma, USA), was used to label exosomes obtained from the conditioned medium. After incubation with the recipient cells for 12 h, fluorescence microscopy was used for imaging.

### Transmission electron microscopy (TEM)

The exosomes were fixed with glutaraldehyde and added dropwise to the copper mesh. 10 μL of 2% phosphotungstic acid solution (pH = 6.5) was added dropwise to the copper mesh and stained for 2 min at room temperature. The exosomes were observed on the TEM (Hitachi, Japan) with an observation voltage of 120 kV to show the morphology of the exosomes and images were acquired.

### Nanoparticle tracking analysis (NTA)

Exosomes were diluted 1000-fold with PBS and the suspension was filtered through a 0.22 μm filter to separate the exosomes from the larger particles. The sample is injected into the cuvette of the ZetaView PMX120 instrument. Set the sample name, save the path and select the corresponding program in the software and perform the test. Use the corresponding software ZetaView 8.05.14 (Particle Metrix, Germany) to analyze the data and generate a report.

### Flow cytometry

The cells (1 × 10^6^) were washed twice with cell staining buffer and were subsequently fixed using fixation buffer (Biolegend, USA). The cell membranes were broken using a permeabilization buffer (Biolegend, USA). Cells were then stained with APC-CD68, PE-CD206 and FITC-CD86 antibodies at 4 °C for 2 h. Cells were subsequently suspended in 500 μL cell staining buffer and examined using a BD Accuri C6 flow cytometer (BD Biosciences, USA).

### Conditional medium (CM) preparation

THP-1 cells were treated with PMA at 37 °C for 24 h. Cells were then treated with different sets of exosomes at 37 °C for 48 h. Cells were then cultured in a serum-free medium at 37 °C for 24 h. The CM was directly used for assays or stored at −80 °C. The CM was filtered and 2% FBS was added.

### Statistical analysis

GraphPad Prism 8.0 and SPSS 26.0 software were used to analyze the data. *χ*^2^ test to analyze the relationship between lncRNA HAGLROS and clinicopathological parameters of patients. The two-tailed unpaired Student’s *t*-test was used to compare the mean values of the two groups. Two-way ANOVA was used to compare the mean values of multiple groups. The Bonferroni test was used for two-way comparison between groups. All experiments were repeated in triplicate and their mean values are presented as the mean ± SD. *P* < 0.05 was considered to indicate a statistically significant difference. The main figures were assembled in Adobe Illustrator and Graphical Abstract diagram by Figdraw.

## Results

### LncRNA HAGLROS is upregulated in breast cancer tissues and associated with poor patient prognosis

**Fig. 1:**
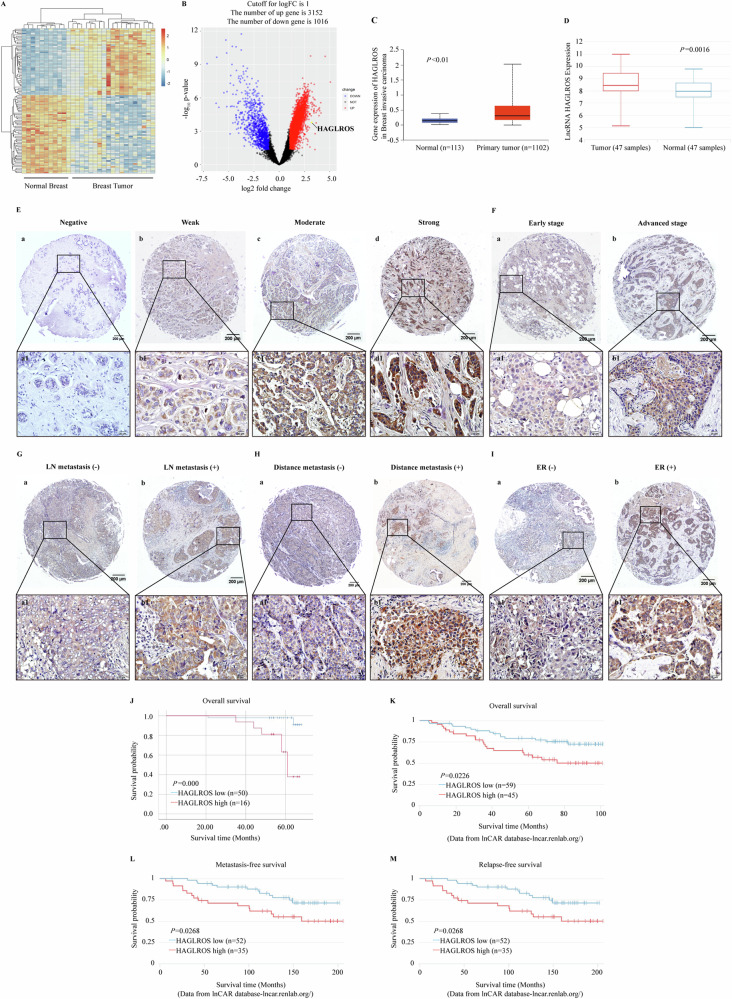
LncRNA HAGLROS upregulation is associated with advanced progression and poor prognosis in breast cancer. **A** Heat map showing the top differentially expressing lncRNAs in breast cancer samples compared to normal tissues (log_2_ |FC | > 1 and *P* < 0.05). **B** Volcano plot showing the expression profiles of lncRNAs. **C**, **D** The expression of lncRNA HAGLROS expression in normal and breast cancer tissues was searched by UALCAN and lnCAR databases. **E** The expression of lncRNA HAGLROS in breast cancer tissues was detected by ISH staining. (a) LncRNA HAGLROS was negative staining in normal breast tissues. (b-d) Weak, moderate, and strong expression of lncRNA HAGLROS in breast cancer tissues (original magnification, a–d: 40 ×; a1–d1: 400 × ). **F** Representative ISH images of lncRNA HAGLROS expression (brown) in different TNM stages of breast cancer tissues (original magnification, a–b: 40×; a1–b1: 400×). **G** Representative ISH images of lncRNA HAGLROS expression (brown) in breast cancer tissues with and without LN metastasis (original magnification, a-b: 40×; a1-b1: 400×). **H** Representative ISH images of lncRNA HAGLROS expression (brown) in breast cancer tissues with and without distant metastasis (original magnification, a-b: 40×; a1-b1: 400×). **I** Representative ISH images (brown) of lncRNA HAGLROS expression in breast cancer tissues with and without ER expression (original magnification, a-b: 40×; a1-b1: 400×). **J** Kaplan-Meier plotter survival analysis was performed to assess the impact of lncRNA HAGLROS on overall survival in breast cancer patients. **K**, **M** The lnCAR database analyzes the effect of lncRNA HAGLROS on overall survival, Metastasis-free survival and Relapse-free survival in breast cancer patients.

To identify important lncRNA that may be involved in breast cancer progression, a total of 3152 upregulated lncRNAs and 1016 downregulated lncRNAs (log_2_ |FC| > 1 and *P* < 0.05) were analyzed by R language to the public database GSE119233 (including 10 normal tissues and 20 breast cancer tissues) for double clustering, and plotting heat and volcano maps. In this study, we focused on the upregulated lncRNAs because of their possible use as therapeutic targets or prognostic biomarkers, among which lncRNA HAGLROS was one of the lncRNA significantly upregulated in breast cancer tissues (Fig. 1A, B). LncRNA HAGLROS is located in human 2q31.1 and consists of 2 exons with a full length of 699 bp (Supplementary Fig. 1A). The sequence of the full-length lncRNA HAGLROS and its minimal free energy (MFE)-based secondary structure are shown in Supplementary Fig. 1B, C. In addition, using an open reading frame (ORF) finder and a conserved domain database, we found that lncRNA HAGLROS encodes a protein of very low potential, which is in agreement with the results of five different online metrics (Supplementary Fig. 1D–F). UALCAN and lnCAR databases found that the expression of lncRNA HAGLROS in breast cancer tissues was significantly higher than that in adjacent normal tissues (Fig. 1C, D).

ISH staining showed that lncRNA HAGLROS expression was upregulated in breast cancer tissues compared with the normal tissues and was mainly localized in the cytoplasm (Fig. 1E). Combined with the results of pathologist scoring, the positive rate of lncRNA HAGLROS was 82.8% (82/99, *P* < 0.001) and strongly positive rate was 36.4% (36/99, *P* < 0.001), both significantly higher than normal tissues (positive rate 26.6%, 21/79; strongly positive rate 2.5%, 2/79) **(**Supplementary Table 4**)**. Furthermore, the clinicopathological analysis revealed that the expression level of lncRNA HAGLROS was positively correlated with lymph node metastasis (*P* = 0.021), distant metastasis (*P* = 0.002), TNM stage (*P* = 0.000) and ER (*P* = 0.038) expression of patients (Fig. 1F-I, Supplementary Table 5). Kaplan-Meier Plotter and lnCAR database survival analysis found that overall survival (*P* = 0.000, *P* = 0.0226), metastasis-free survival (*P* = 0.0268), and relapse-free survival (*P* = 0.0268) were significantly higher in breast cancer patients with low lncRNA HAGLROS expression than in lncRNA HAGLROS high expression patients (Fig. 1J–M). Univariate Cox regression model analysis showed that distant metastasis (*P* = 0.003), TNM stage (*P* = 0.000) and lncRNA HAGLORS expression level (*P* = 0.002) were strongly associated with poor prognosis in breast cancer patients, and multivariate Cox regression model analysis showed that lncRNA HAGLROS expression level (*P* = 0.048) was an independent prognostic risk factor for patients (Supplementary Table 6). Taken together, the upregulation of lncRNA HAGLROS was significantly associated with the poor prognosis of breast cancer patients.

### LncRNA HAGLROS promotes proliferation, metastasis, EMT progression and angiogenesis in breast cancer cells

**Fig. 2:**
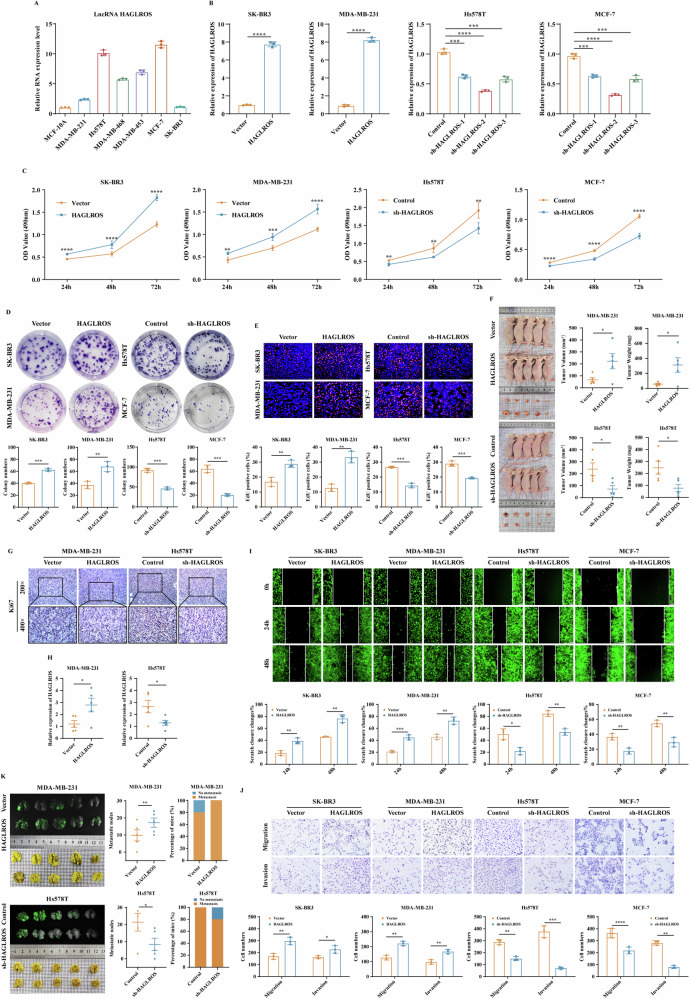
LncRNA HAGLROS promotes proliferation and metastasis of breast cancer cells in vitro and in vivo. **A** The expression of lncRNA HAGLROS in breast epithelial cells and breast cancer cells was detected by qRT-PCR assay. GAPDH was used as an internal control. **B** The vector and lncRNA HAGLROS overexpression plasmid were transfected in SK-BR3 and MDA-MB-231 cells, and control, sh-lncRNA HAGLROS-1, sh-lncRNA HAGLROS-2, sh-lncRNA HAGLROS-3 silencing plasmids were transfected in Hs578T and MCF-7 cells (lncRNA HAGLROS overexpression group compared with vector, sh-lncRNA HAGLROS group compared with control), and the transfection effect of lncRNA HAGLROS was detected by qRT-PCR assay. GAPDH was used as an internal control. **C**–**E** The effect of differentially expressing lncRNA HAGLROS on the proliferation ability of breast cancer cells was detected by MTT, colony formation and EdU assays. **F** Representative images of xenograft tumors in nude mice with breast cancer cells differentially expressing lncRNA HAGLROS (upper: vector and lncRNA HAGLROS overexpression groups; lower: control and lncRNA HAGLROS silenced group), and the volume and weight of xenograft tumors (*n* = 5 per group). **G** The expression of Ki67 in the xenograft tumor tissues was detected by IHC staining. **H** The expression of lncRNA HAGLROS in the xenograft tumor tissues was detected by qRT-PCR assay. **I**, **J** The effect of differentially expressing lncRNA HAGLROS on the migration and invasion ability of breast cancer cells was detected by wound healing and Transwell assays. **K** Breast cancer cells differentially expressing lncRNA HAGLROS were separately injected via tail vein into nude mice for in vivo metastasis (*n* = 5 per group). Representative images show the number of metastatic nodules in the lungs (left), and the number of nude mice that developed lung metastases (right).

To determine the biological function of lncRNA HAGLROS in breast cancer cells, we examined lncRNA HAGLROS expression in breast cancer cells, and qRT-PCR results showed that the expression of lncRNA HAGLROS was significantly higher in breast cancer cell lines than in breast epithelial cells **(**Fig. 2A**)**. The low expressing cell lines SK-BR3 and MDA-MB-231 were lentivirus transfected to construct lncRNA HAGLROS overexpression stable cell lines, and the high expressing cell lines Hs578T and MCF-7 were lentivirus transfected to construct three lncRNA HAGLROS silencing stable cell lines. The transfection effect was verified by qRT-PCR, and the sh-lncRNA HAGLROS-2 sequence with the best silencing effect was selected for functional experiments (Fig. 2B).

To clarify the effect of lncRNA HAGLROS on the proliferation ability of breast cancer cells, we performed MTT, colony formation and EdU assays on breast cancer cells differentially expressing lncRNA HAGLROS. The results showed that lncRNA HAGLROS overexpression significantly promoted breast cancer cell growth, clone formation and DNA replication ability, while the silencing of lncRNA HAGLROS group had the opposite effect (Fig. 2C–E). To further verify the results of our in vitro study, we investigated the effect of lncRNA HAGLROS on tumorigenesis using a xenograft model. Tumor volume and weight were significantly higher in the lncRNA HAGLROS overexpression group than in the control group, while the silencing of lncRNA HAGLROS group had the opposite effect (Fig. 2F). In addition, IHC staining and qRT-PCR assays on tumor sections and fresh tissues of nude mice showed that the number of Ki67-positive cells and the expression level of lncRNA HAGLROS in lncRNA HAGLROS overexpressing tissues were significantly higher than those in the control group, while the number of Ki67-positive cells in lncRNA HAGLROS low-expressing tissues and the expression levels of lncRNA HAGLROS were significantly lower than those of the control group (Fig. 2G, H). These results suggest that lncRNA HAGLROS plays an important tumorigenic role in breast cancer.

**Fig. 3:**
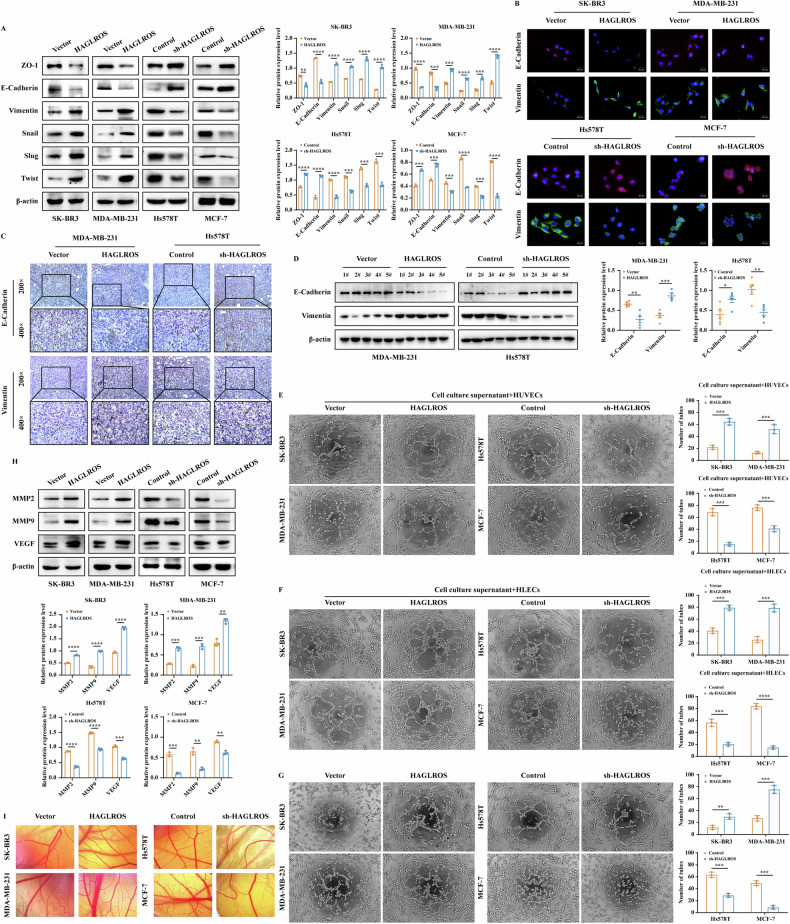
LncRNA HAGLROS promotes the EMT process and angiogenesis of breast cancer cells in vitro and in vivo. **A** The protein expression levels of EMT-related markers in breast cancer cells differentially expressing lncRNA HAGLROS were detected by western blotting assay. β-actin was used as an internal control. **B** The fluorescence expression levels of E-Cadherin and Vimentin in breast cancer cells differentially expressing lncRNA HAGLROS were detected by IF staining. **C**, **D** The expression of E-Cadherin and Vimentin in the xenograft tumor tissues was detected by IHC staining and western blotting assay. **E**, **F** HUVECs or HLECs were cultured with the supernatants of breast cancer cells differentially expressing lncRNA HAGLROS and the microtubule formations were detected by endothelial tube formation assay. **G** The effect of differentially expressed lncRNA HAGLROS on the angiogenesis ability of breast cancer cells was detected by VM assay. **H** The protein expression levels of angiogenesis-related markers in breast cancer cells differentially expressing lncRNA HAGLROS were detected by western blotting assay. β-actin was used as an internal control. **I** The effect of breast cancer cells differentially expressing lncRNA HAGLROS on angiogenesis in vitro was examined by CAM assay.

Subsequently, we investigated the function of the lncRNA HAGLROS in breast cancer cell metastasis. Wound healing and Transwell assays showed that lncRNA HAGLROS overexpression significantly promoted the migration and invasion ability of breast cancer cells, and vice versa (Fig. 2I, J). A breast cancer lung metastasis model was constructed, in vivo fluorescence imaging and gross observation of lung tissue further confirmed that lncRNA HAGLROS overexpression significantly promoted lung metastatic ability, and vice versa (Fig. 2K). To further investigate the relationship between lncRNA HAGLROS promotion of breast cancer metastasis and EMT. Western blot results showed that lncRNA HAGLROS overexpression significantly downregulated the protein expression levels of the epithelial markers ZO-1 and E-Cadherin, and upregulated the protein expression levels of the mesenchymal markers Vimentin, Snail, Slug and Twist, and vice versa (Fig. 3A). IF, IHC showed that lncRNA HAGLROS overexpression downregulated the expression levels of E-Cadherin and upregulated the expression levels of Vimentin, and vice versa (Fig. 3B–D). These results suggest that lncRNA HAGLROS overexpression promotes the metastasis and EMT process of breast cancer.

To clarify the effect of lncRNA HAGLROS on angiogenesis in breast cancer cells, endothelial tube formation and VM results showed that lncRNA HAGLROS overexpression significantly promoted neovascularization and luminal structure formation ability in HUVECs, HLECs and breast cancer cells, and vice versa (Fig. 3E–G). Western blotting results showed that lncRNA HAGLROS overexpression significantly upregulated the protein expression levels of MMP2, MMP9 and VEGF, and vice versa (Fig. 3H). CAM results showed that lncRNA HAGLROS overexpression significantly promoted the formation of neovascularization and branching in chick embryos, and vice versa (Fig. 3I). These findings suggest that lncRNA HAGLROS promotes breast cancer metastasis by regulating angiogenesis.

### LncRNA HAGLROS as a sponge for miR-135b-3p regulates the malignant evolution of breast cancer

**Fig. 4:**
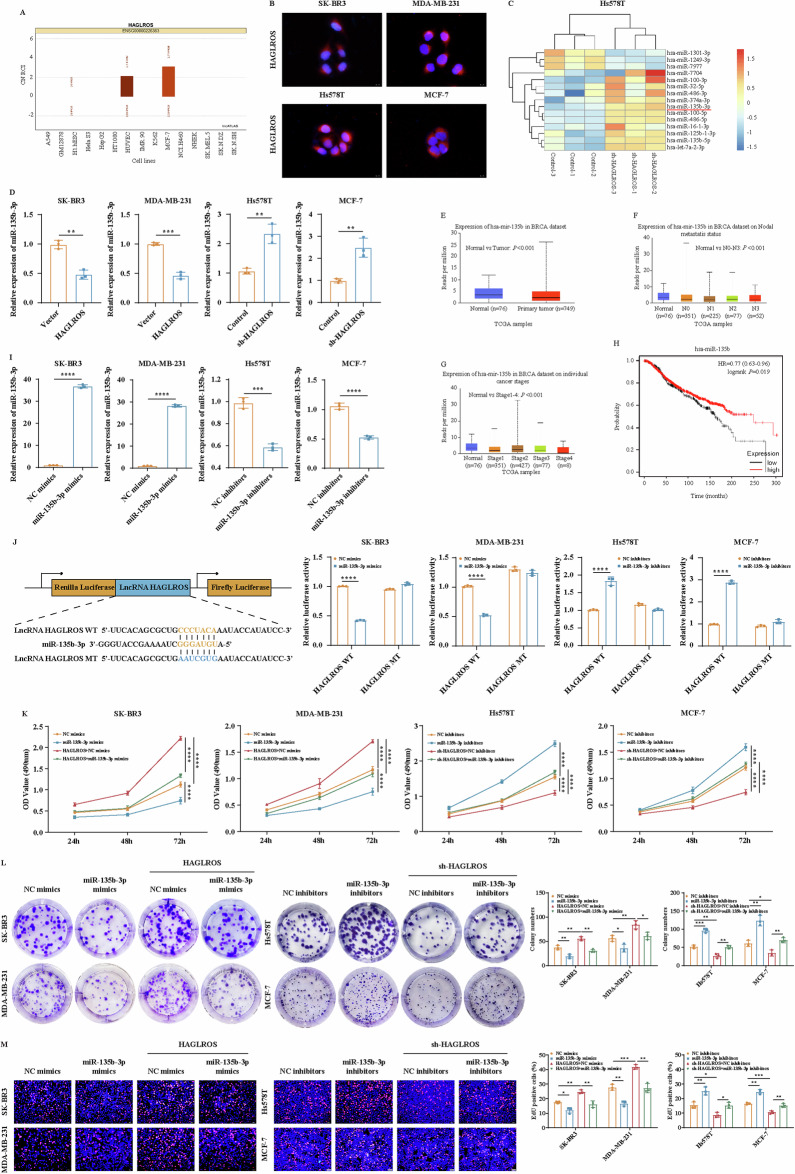
LncRNA HAGLROS as a sponge for miR-135b-3p promotes the proliferative ability of breast cancer cells. **A**, **B** The localization of lncRNA HAGLROS in breast cancer cells was determined by the lncATLAS database and FISH assay. **C** Heat map representation of differentially expressed miRNA after silencing of lncRNA HAGLROS in Hs578T cells (log_2_ |FC| > 1 and *P* < 0.05). **D** The expression level of miR-135b-3p in breast cancer cells differentially expressing lncRNA HAGLROS was detected by qRT-PCR assay. U6 was used as an internal control. **E** The expression level of miR-135b in normal and breast cancer tissues was searched by the UALCAN database. **F**, **G** The correlation of miR-135b expression levels in breast cancer with lymph node metastasis and TNM stage were searched by the UALCAN database. **H** Kaplan-Meier plotter survival analysis was performed to assess the impact of miR-135b on overall survival in breast cancer patients. **I** The transfection effect of breast cancer cells transfected with miR-135b-3p mimics and inhibitors was detected by qRT-PCR assay. U6 was used as an internal control. **J** Breast cancer cells co-transfected with wild-type or mutant lncRNA HAGLROS and miR-135b-3p or control were detected by dual luciferase reporter assay. **K**–**M** The effect of co-transfected lncRNA HAGLROS and miR-135b-3p on the proliferation ability of breast cancer cells was determined by MTT, colony formation and EdU assays.

LncRNA has been reported to act as ceRNA to regulate miRNA expression and biological functions. The regulatory mode of lncRNA is related to its subcellular localization. LncATLAS database and FISH results showed that lncRNA HAGLROS was mainly localized in the cytoplasm of breast cancer cells (Fig. 4A, B). To further explore the molecular mechanisms by which lncRNA HAGLROS regulates the malignant evolution of breast cancer, RNA sequencing was performed to identify miRNA regulated by lncRNA HAGLROS and the results revealed that only miR-135b-3p had binding sites to lncRNA HAGLROS (Fig. 4C). qRT-PCR results showed that the expression of miR-135b-3p was significantly higher in breast epithelial cells than in breast cancer cell lines (Supplementary Fig. 2A). qRT-PCR results showed that silencing of lncRNA HAGLROS significantly upregulated the expression level of miR-135b-3p compared to the control group, and vice versa (Fig. 4D). UALCAN database revealed that miR-135b-3p expression was significantly lower in breast cancer tissues than in normal tissues and negatively correlated with TNM stage and lymph node metastasis (Fig. 4E–G). Kaplan-Meier plotter database showed that breast cancer patients with low miR-135b-3p expression had significantly lower survival than those with high miR-135b-3p expression, suggesting that miR-135b-3p plays a suppressor role in the malignant evolution of breast cancer (Fig. 4H). SK-BR3 and MDA-MB-231 cells were transfected with NC mimics and miR-135b-3p mimics, and Hs578T and MCF-7 cells were transfected with NC inhibitors and miR-135b-3p inhibitors. The transfection efficiency was verified by qRT-PCR (Fig. 4I). Dual luciferase reporter results showed that miR-135b-3p overexpression significantly reduced the luciferase activity of lncRNA HAGLROS-WT vector, but failed to reduce the luciferase activity of the mutant vector, and vice versa (Fig. 4J). The above results suggest that lncRNA HAGLROS interacts with miR-135b-3p, and lncRNA HAGLROS can act as ceRNA to adsorb miR-135b-3p.

**Fig. 5:**
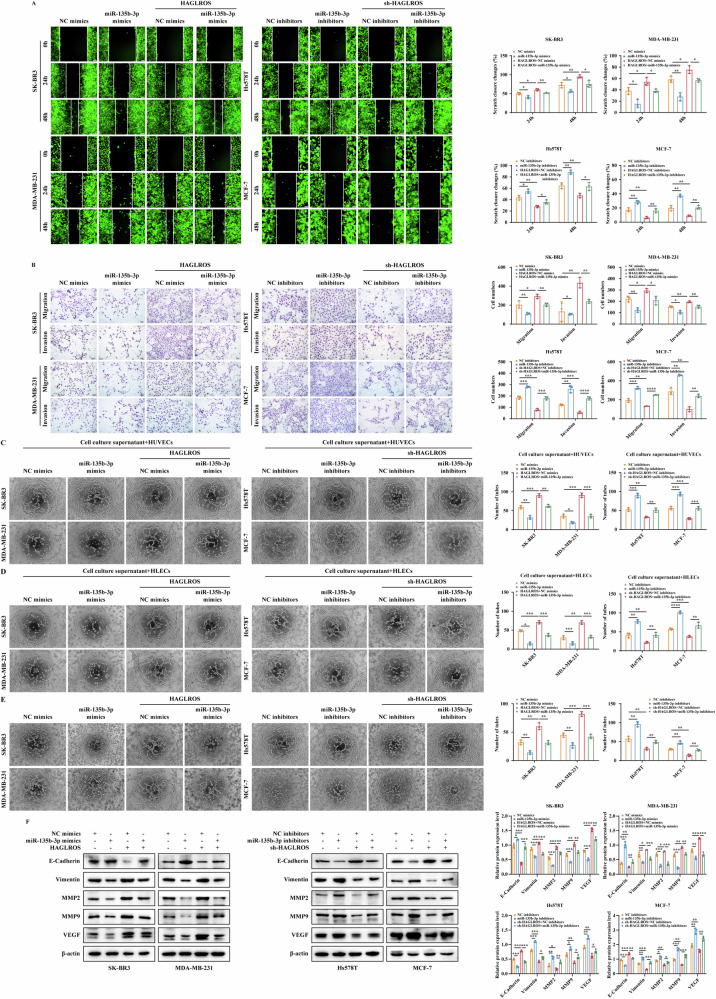
LncRNA HAGLROS as a sponge for miR-135b-3p promotes the migration and invasion ability of breast cancer cells. **A**, **B** The effect of co-transfected lncRNA HAGLROS and miR-135b-3p on the migration and invasion ability of breast cancer cells was determined by wound healing and Transwell assays. **C**, **D** HUVECs or HLECs were cultured with the supernatants of breast cancer cells co-transfected with lncRNA HAGLROS and miR-135b-3p, and the microtubule formations were detected by endothelial tube formation assay. **E** The effect of co-transfected lncRNA HAGLROS and miR-135b-3p on the angiogenesis ability of breast cancer cells was detected by VM assay. **F** The protein expression levels of EMT and angiogenesis-related markers in breast cancer cells co-transfected with lncRNA HAGLROS and miR-135b-3p were detected by western blotting assay. β-actin was used as an internal control.

To elucidate whether the interaction between lncRNA HAGLROS with miR-135b-3p can regulate the malignant evolution of breast cancer, we performed rescue experiments to examine the effect of miR-135b-3p in lncRNA HAGLROS differentially expressing cells. MTT, colony formation and EdU results showed that miR-135b-3p mimics restored the proliferative capacity of breast cancer cells promoted by lncRNA HAGLROS upregulation, and vice versa (Fig. 4K–M). Wound healing and Transwell results showed that miR-135b-3p mimics restored the migration and invasive ability of breast cancer cells promoted by lncRNA HAGLROS upregulation, and vice versa (Fig. 5A, B). Endothelial tube formation and VM results showed that miR-135b-3p mimics restored the neovascularization and luminal structure formation abilities of HUVECs, HLECs and breast cancer cells promoted by lncRNA HAGLROS upregulation, and vice versa (Fig. 5C–E). Western blotting results showed that miR-135b-3p mimics restored the expression of EMT and angiogenesis-related markers promoted by lncRNA HAGLROS upregulation, and vice versa (Fig. 5F). These results suggest that lncRNA HAGLROS promotes the proliferation, migration, invasion, EMT process and angiogenesis ability of breast cancer cells by sponging miR-135b-3p.

### LncRNA HAGLROS acts as a sponge for miR-135b-3p to regulate COL10A1 expression and promote the malignant evolution of breast cancer

**Fig. 6:**
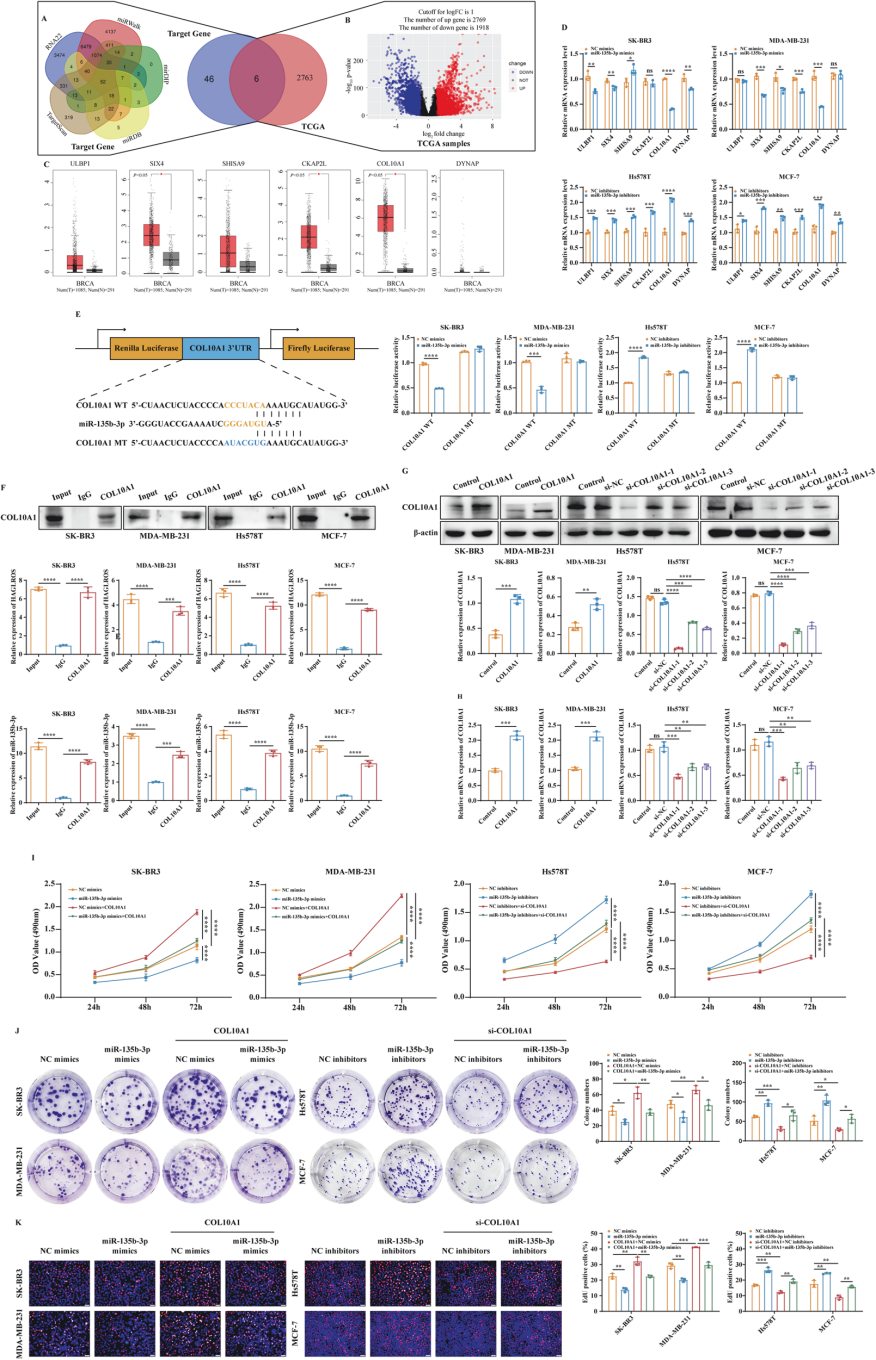
LncRNA HAGLROS acts as a sponge for miR-135b-3p to regulate COL10A1 expression and promotes the proliferative ability of breast cancer cells. **A** The potential target genes of miR-135b-3p were predicted by RNA22, miRWalk, mirDIP, miRDB, and TargetScan databases. **B** Volcano plot showing the expression profile of differential genes in breast cancer (log_2_ |FC| > 1 and *P* < 0.05). **C** The expression level of ULBP1, SIX4, SHISA9, CKAP2L, COL10A1 and DYNAP in normal and breast cancer tissues were searched by the GEPIA database. **D** The mRNA expression level of ULBP1, SIX4, SHISA9, CKAP2L, COL10A1 and DYNAP in breast cancer cells differentially expressing miR-135b-3p was detected by qRT-PCR assay. GAPDH was used as an internal control. **E** Breast cancer cells co-transfected with wild-type or mutant COL10A1 and miR-135b-3p or control were detected by dual luciferase reporter assay. **F** The interaction between lncRNA HAGLROS and miR-135b-3p and COL10A1 was analyzed by RIP assay. **G**, **H** The control and COL10A1 overexpression plasmid were transfected in SK-BR3 and MDA-MB-231 cells, and control, si-COL10A1-1, si-COL10A1-2, si-COL10A1-3 were transfected in Hs578T and MCF-7 cells (COL10A1 overexpression group compared with control, si-COL10A1 group compared with control), and the transfection effect of COL10A1 was detected by western blotting and qRT-PCR assays. **I**–**K** The effect of co-transfected miR-135b-3p and COL10A1 on the proliferation ability of breast cancer cells was determined by MTT, colony formation and EdU assays.

To further investigate the molecular mechanism of lncRNA HAGLROS/miR-135b-3p affecting the malignant evolution of breast cancer, we predicted 52 potential target genes of miR-135b-3p by RNA22, miRWalk, mirDIP, miRDB and TargetScan databases (Fig. 6A). A total of 2769 genes were upregulated in breast cancer tissues (log_2_ |FC| > 1 and *P* < 0.05) by R language to the TCGA database for clustering analysis and plotting volcano maps (Fig. 6B). There were 6 overlapping genes, including ULBP1, SIX4, SHISA9, CKAP2L, COL10A1 and DYNAP (Fig. 6C). qRT-PCR results showed that miR-135b-3p regulated the expression of COL10A1 most significantly (Fig. 6D). Therefore, COL10A1 was selected as the target gene of miR-135b-3p, and the expression of COL10A1 was significantly higher in breast cancer cell lines than in breast epithelial cells (Supplementary Fig. 2B). Dual luciferase reporter results showed that miR-135b-3p mimics significantly reduced the luciferase activity of the COL10A1-WT vector, but failed to reduce the luciferase activity of the mutant vector, and vice versa (Fig. 6E). To confirm the interaction between miR-135b-3p and lncRNA HAGLROS and COL10A1, RIP results showed that COL10A1 antibody could pull down the expression levels of endogenous lncRNA HAGLROS and miR-135b-3p, further validating its binding potential (Fig. 6F). Subsequently, COL10A1 overexpression was constructed by plasmid and COL10A1 silencing sequence was constructed by siRNA. The transfection efficiency was verified by western blotting and qRT-PCR, and the si-COL10A1-1 sequence with the best silencing effect was selected for subsequent functional assays (Fig. 6G, H). These results indicated that miR-135b-3p could regulate the expression level of COL10A1.

**Fig. 7:**
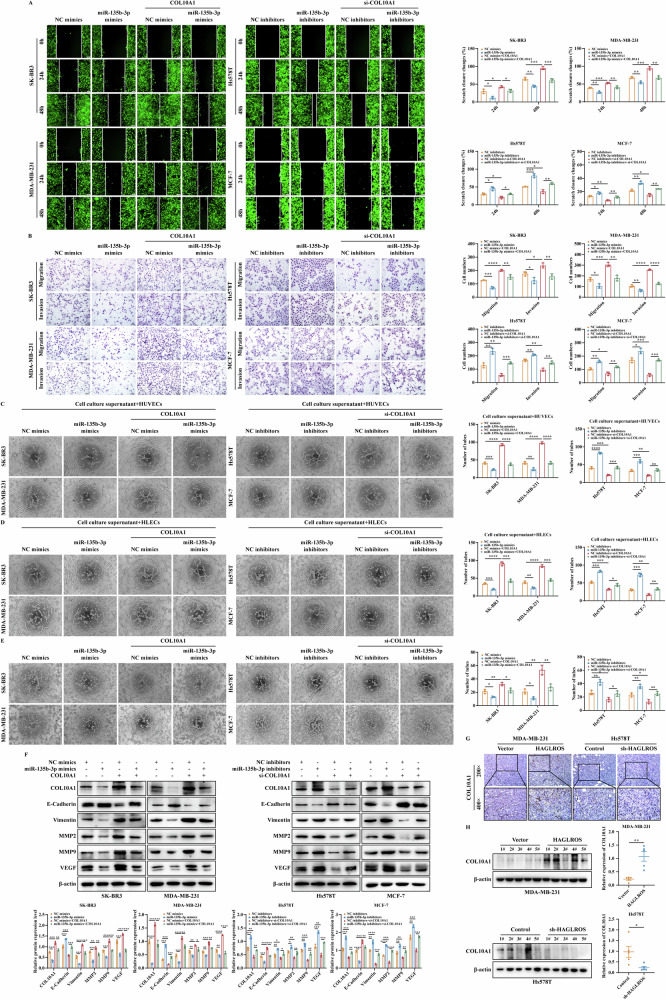
LncRNA HAGLROS acts as a sponge for miR-135b-3p to regulate COL10A1 expression and promotes the migration and invasion ability of breast cancer cells. **A**, **B** The effect of co-transfected miR-135b-3p and COL10A1 on the migration and invasion ability of breast cancer cells was determined by wound healing and Transwell assays. **C**, **D** HUVECs or HLECs were cultured with the supernatants of breast cancer cells co-transfected with miR-135b-3p and COL10A1, and the microtubule formations were detected by endothelial tube formation assay. **E** The effect of co-transfected miR-135b-3p and COL10A1 on the angiogenesis ability of breast cancer cells was detected by VM assay. **F** The protein expression levels of EMT and angiogenesis-related markers in breast cancer cells co-transfected with miR-135b-3p and COL10A1 were detected by western blotting assay. β-actin was used as an internal control. **G**, **H** The expression of COL10A1 in the xenograft tumor tissues was detected by IHC staining and western blotting assays.

To elucidate whether the interaction between miR-135b-3p with COL10A1 can regulate the malignant evolution of breast cancer, we performed rescue experiments to examine the effect of COL10A1 in miR-135b-3p differentially expressing cells. MTT, colony formation and EdU results showed that COL10A1 restored the proliferation ability of breast cancer cells suppressed by miR-135b-3p upregulation, and vice versa (Fig. 6I–K). Wound healing and Transwell results showed that COL10A1 restored the migration and invasive ability of breast cancer cells suppressed by miR-135b-3p upregulation, and vice versa (Fig. 7A, B). Endothelial tube formation and VM results showed that COL10A1 restored the ability of miR-135b-3p upregulation-suppressed angiogenesis and luminal structure formation in HUVECs, HLECs and breast cancer cells, and vice versa (Fig. 7C–E). Western blotting results showed that COL10A1 restored the ability of miR-135b-3p upregulation-suppressed the expression of EMT and angiogenesis-related markers, and vice versa (Fig. 7F). IHC and western blotting results showed that lncRNA HAGLROS overexpression upregulated COL10A1 protein expression levels in nude mice tissues and vice versa (Fig. 7G, H). These results suggest that lncRNA HAGLROS/miR-135b-3p axis regulates the proliferation, migration, invasion, EMT process and angiogenesis ability of breast cancer cells by targeting COL10A1.

### Breast cancer cell-derived exosomal lncRNA HAGLROS induces TAM/M2 polarization through the p-STAT3 signaling pathway

**Fig. 8:**
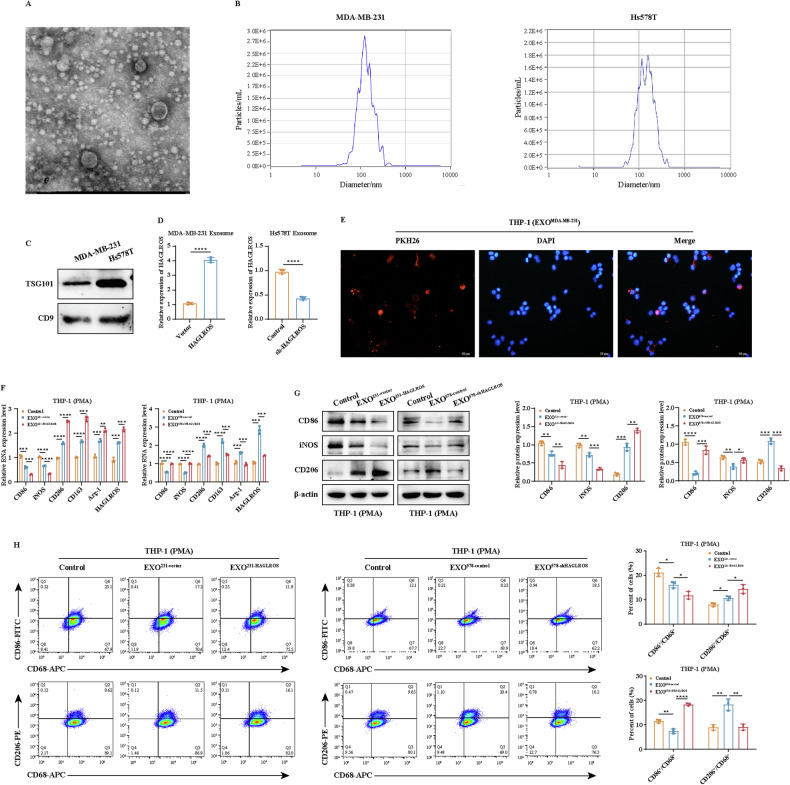
Exosomal lncRNA HAGLROS promotes TAM/M2 polarization. **A**, **B** Exosomes isolated from supernatants of breast cancer cells were detected by TEM and NTA. **C** The expression of exosome markers TSG101 and CD9 was detected by western blotting assay. **D** The expression of lncRNA HAGLROS in exosomes derived from breast cancer cells differentially expressing lncRNA HAGLROS was detected by qRT-PCR assay. **E** Representative fluorescence microscopy showing the uptake of PKH26-labeled exosomes (red fluorescent dye) derived from MDA-MB-231 cells by recipient macrophages. **F**–**H** Macrophages were cultured with exosomes derived from breast cancer cells differentially expressing lncRNA HAGLROS and the expression of TAM/M1, TAM/M2-related markers and lncRNA HAGLROS in macrophages was detected by qRT-PCR, western blotting and flow cytometry assays.

To explore the potential mechanism of association between lncRNA HAGLROS and breast cancer microenvironment, exosomes were isolated from the supernatant of breast cancer cells for validation. qRT-PCR results showed that the expression of lncRNA HAGLROS was significantly higher in breast cancer cell exosomes than in breast epithelial cell exosomes (Supplementary Fig. 2C).TEM observed that the exosomes were about 100 nm in diameter with a saucer-like structure (Fig. 8A). NTA results showed that the exosome particles were 126.8 nm in diameter (Fig. 8B). Western blot results showed that the exosome markers TSG101 and CD9 were expressed in exosomes derived from breast cancer cells (Fig. 8C). To clarify whether lncRNA HAGLROS can be wrapped into exosomes, we extracted exosomes from supernatants of breast cancer cells differentially expressing lncRNA HAGLROS. qRT-PCR results showed that the expression of lncRNA HAGLORS was significantly upregulated in exosomes overexpressing lncRNA HAGLROS compared to controls, and vice versa (Fig. 8D). These results suggest that lncRNA HAGLROS is packaged into exosomes. To explore whether exosomes can be internalized into macrophages and regulate TAMs polarization, PKH26-labeled exosomes (10 μg/mL) were incubated with THP-1 cells, and fluorescence microscopy showed that PKH26-labeled exosomes could be internalized by THP-1 (Fig. 8E). Subsequently, THP-1 cells were co-cultured with exosomes isolated from breast cancer cells differentially expressing lncRNA HAGLROS. qRT-PCR, Western blotting and flow cytometry results showed that lncRNA HAGLROS overexpressing exosomes significantly downregulated the expression levels of TAM/M1 markers (CD86 and iNOS) compared to controls, while upregulating the expression of lncRNA HAGLROS and TAM/M2 markers (CD206, CD163, Arg-1), and vice versa (Fig. 8F–H). To further validate the results of the in vitro experiments, we collected breast cancer plasma exosomes and breast cancer tissues for qRT-PCR and IHC experiments, which showed that the expression of lncRNA HAGLROS was higher in breast cancer plasma exosomes than that of plasma exosomes from healthy individuals, and that the expression of CD86 in breast cancer tissues was lower than that in paracancerous tissues, while the expression of CD206 was in the opposite direction (Supplementary Fig. 3A, B). These results suggest that the exosomal lncRNA HAGLROS derived from breast cancer cells inhibits TAM/M1 polarization and promotes TAM/M2 polarization.

To further investigate the molecular mechanism by which exosomal lncRNA HAGLROS regulates TAM/M2 polarization, we investigated several immune-related signaling pathways, including CEBPβ, MAPK, and the STAT signaling pathway, which are all involved in TAM/M2 polarization. Western blotting results showed that exosomal lncRNA HAGLROS had no effect on the protein expression levels of p-ERK, p-STAT6, and CEBPβ. However, the expression level of p-STAT3 was significantly up-regulated after treatment of exosomes with high expression of lncRNA HAGLROS (Supplementary Fig. 3C). Subsequently, macrophages were treated with different concentrations of STAT3 inhibitors and agonists. The expression of pSTAT3 and STAT3 was detected by western blotting assay, and the 0.8 μM STAT3 inhibitor and 4 nM agonist were screened for subsequent experiments (Supplementary Fig. 3D). Western blotting results showed that STAT3 inhibitors restored the expression of TAM/M2-associated markers promoted by the up-regulation of exosomal lncRNA HAGLROS, and vice versa (Supplementary Fig. 3E). These results suggest that the breast cancer cell-derived exosomal lncRNA HAGLROS promotes TAM/M2 polarization through activation of the p-STAT3 signaling pathway.

### Exosomal lncRNA HAGLROS promotes malignant progression of breast cancer by inducing TAM/M2 polarization

To elucidate the effect of exosomal lncRNA HAGLROS-mediated TAM/M2 polarization on breast cancer cells, exosomes isolated from breast cancer cells differentially expressing lncRNA HAGLROS were treated with THP-1, and the supernatant was subsequently collected to act on breast cancer cells, HUVECs and HLECs. MTT and EdU results showed that THP-1 treated with exosomes overexpressing lncRNA HAGLROS significantly promoted the proliferation and DNA replication ability of breast cancer cells, and vice versa (Supplementary Fig. 4A, B). Wound healing and Transwell results showed that THP-1 treated with exosomes overexpressing lncRNA HAGLROS significantly promoted the migration and invasive ability of breast cancer cells, and vice versa (Supplementary Fig. 4C, D). Endothelial tube formation and VM assays showed that THP-1 treated with exosomes overexpressing lncRNA HAGLROS significantly promoted neovascularization and luminal structure formation ability of HUVECs, HLECs and breast cancer cells, and vice versa (Supplementary Fig. 4E). Western blot results showed that THP-1 treated with exosomes overexpressing lncRNA HAGLROS significantly upregulated the expression of EMT and angiogenesis-related markers in breast cancer cells, and vice versa (Supplementary Fig. 4F). A xenograft tumor model was constructed to validate the above in vitro results, and MDA-MB-231 cells and THP-1 cells treated with overexpressed lncRNA HAGLROS exosomes were co-injected subcutaneously into nude mice, and the tumor growth was examined. The results showed that exosomal lncRNA HAGLROS-treated THP-1 cells significantly increased the tumor volume and weight (Supplementary Fig. 4G). In addition, IHC staining of tumor sections showed that exosomal lncRNA HAGLROS-treated THP-1 cells significantly decreased the expression of CD86 in tumor tissues, while increasing the expression of CD206 (Supplementary Fig. 4H). These results suggest that the exosomal lncRNA HAGLROS promotes TAM/M2 polarization through the p-STAT3 signaling pathway and enhances malignant evolution in breast cancer.

## Discussion

LncRNA has been considered as “junk” formed during transcription in the past decades, and there are approximately twice as many human lncRNA as protein-coding genes. lncRNA are still at a relatively early stage of nomenclature, classification and identification compared to the widely studied miRNA and protein-coding genes [24]. There is growing evidence that lncRNA plays important roles in both physiological and pathological processes, such as cell cycle, cell differentiation and tumorigenesis as well as other processes, including epigenetics and chromatin remodeling [25, 26]. Several studies have shown that lncRNA is closely associated with the development of various malignancies, including liver, pancreatic and breast cancers, and can be an independent risk factor for poor clinical prognosis [27–29]. Therefore, elucidating the role of lncRNA in tumors may help to understand tumor pathogenesis and reveal new therapeutic targets. LncRNA HAGLROS, a novel lncRNA, was first reported in gastric cancer in 2018 [30]. Several studies subsequently confirmed that lncRNA HAGLROS was upregulated in the expression of various tumors including osteosarcoma, ovarian cancer, intrahepatic cholangiocarcinoma, nephroblastoma, lung cancer and hepatocellular carcinoma, and regulated tumor proliferation, metastasis and EMT processes [13, 16, 31–34]. In this study, we found that high expression of lncRNA HAGLROS was associated with lymph node metastasis, distant metastasis, TNM stage and ER expression in breast cancer patients, and was associated with shorter overall survival in breast cancer patients, which could be an independent risk factor for poor prognosis in breast cancer patients. In addition, we demonstrated that lncRNA HAGLROS promotes the proliferation, metastasis, EMT process and angiogenic ability of breast cancer cells. The above suggests that lncRNA HAGLROS is highly expressed in breast cancer and promotes the malignant evolution of breast cancer.

The different localization of lncRNA in the cell determines their role in tumor progression. In the nucleus, lncRNA regulates the transcriptional program through chromatin interactions and remodeling, and establishes the spatial organization of the nuclear compartment through scaffolding. In the cytoplasm, lncRNA mediates signal transduction pathways and post-transcriptional regulation of gene expression through multiple pathways, including ceRNA, mRNA stabilization and translational regulation) [35]. To investigate the localization of lncRNA HAGLROS in breast cancer, we confirmed that lncRNA HAGLROS was mainly localized in the cytoplasm of breast cancer cells, with a small amount in the nucleus. Therefore, the lncRNA HAGLROS may act as a ceRNA adsorbing miRNA in breast cancer cells. Several studies have shown that the lncRNA HAGLROS acts as a miRNA sponge regulating the expression levels of target genes in a variety of tumors. For example, lncRNA HAGLROS promotes the proliferation, migration and invasive ability of diffuse large B-cell lymphoma cells by sponging miR-100 [36]. LncRNA HAGLROS acts as a molecular sponge for miR-26b-5p to promote proliferation and inhibit apoptosis of ovarian cancer cells [37]. To investigate which miRNA is adsorbed by the lncRNA HAGLROS in breast cancer cells, we sequenced the transcriptome of breast cancer cells with silenced lncRNA HAGLROS, and the upregulated miR-135b-3p was screened and confirmed by relevant assays that lncRNA HAGLROS has binding potential with miR-135b-3p in breast cancer cells. Studies have shown that miR-135b-3p is a tumor suppressor that is downregulated in a variety of tumors and correlates with poor prognosis of patients [38]. Our study confirmed that lncRNA HAGLROS regulates the proliferation, migration, invasion, EMT process and angiogenesis ability of breast cancer cells through miR-135b-3p. It is suggested that lncRNA HAGLROS can act as a molecular sponge for miR-135b-3p to promote the malignant evolution of breast cancer.

It is well known that miRNA regulate gene expression by directly binding to the 3’-UTR of target gene mRNAs, promoting their degradation or inhibiting their translation. LncRNA indirectly regulates gene expression by competitively inhibiting the binding of miRNA to target gene mRNA through the adsorption of miRNA, thereby affecting tumor development. To investigate the molecular mechanism of lncRNA HAGLROS/miR-135b-3p regulating the malignant progression of breast cancer, we screened the target gene COL10A1 of miR-135b-3p by database prediction and confirmed the binding potential of miR-135b-3p to COL10A1 by relevant assays. As a major component of the stroma, COL10A1 is involved in different biological behaviors of tumors. Studies have shown that COL10A1 is highly expressed in a variety of tumor tissues and plays an important role as a regulatory hub in the evolution of malignancy. For example, miR-26a-5p reduces COL10A1 expression by binding to the 3’-UTR of COL10A1, thereby inhibiting the proliferation, migration and invasive ability of gastric cancer cells [39]. miR-384 inhibits autophagy and promotes apoptosis in non-small cell lung cancer cells through the downregulation of COL10A1 expression [40]. Our study confirmed that miR-135b-3p regulates the proliferation, migration, invasion, EMT process and angiogenic ability of breast cancer cells through COL10A1. The above suggests that lncRNA HAGLROS promotes the malignant evolution of breast cancer through the miR-135b-3p/COL10A1 axis.

TAMs are the most abundant cell population in the tumor stroma and are an important component of the tumor immune microenvironment. Various stimuli in TME polarize TAMs into TAM/M1 and TAM/M2 phenotypes, which play a dual role in tumor immunity, both promoting and inhibiting tumor growth [41]. Exosome-packaged lncRNA are involved in the interactive “dialog” between TAMs and tumor cells, thus promoting tumor development [42]. The characteristics of exosomes in the immune response depend in part on their origin, and exosomes from immune cells or tumors exert different biological effects depending on their composition [43]. Tumor-derived exosomal lncRNA promotes TAMs polarization, while TAMs-derived exosomal lncRNA affects tumor cell proliferation, metastasis, angiogenesis, and chemoresistance [44]. For example, renal cancer cell-derived exosomes promote tumor development by transferring lncRNA ARSR to induce TAM/M2 polarization [45]. Pancreatic cancer-derived exosomes promote the proliferation and metastasis of pancreatic cancer cells by inducing TAM/M2 polarization through the transfer lncRNA FGD5-AS1 [46]. TAMs-derived exosomes transfer lncRNA MMPA to tumor cells and activate glycolytic pathways to promote the malignant development of hepatocellular carcinoma [47]. In conclusion, although exosomal lncRNA from donor cells are involved in various biological functions of recipient cells, further studies are needed to discover other exosomal lncRNA involved in TAMs polarization and tumor progression. In this study, the exosome-derived lncRNA HAGLROS from breast cancer cells induced TAM/M2 polarization through activation of the p-STAT3 signaling pathway, which in turn promoted breast cancer cell proliferation, migration, invasion, EMT process, and angiogenesis.

Taken together, lncRNA HAGLROS is highly expressed in breast cancer and predicts poor patient prognosis, and promotes the biological behavior of breast cancer cells. LncRNA HAGLROS promotes proliferation, migration, invasion, EMT process and angiogenesis of breast cancer cells by targeting the miR-135b-3p/COL10A1 axis. Meanwhile, breast cancer cell-derived exosomal lncRNA HAGLROS induces TAM/M2 polarization through the p-STAT3 pathway and enhances malignant evolution of breast cancer cells. This study elucidated that lncRNA HAGLROS/miR-135b-3p/COL10A1 signaling axis is closely related to breast cancer development, and that exosomes play an important role in breast cancer TME by transferring lncRNA HAGLROS, which is expected to be a novel molecular marker for breast cancer diagnosis and prognosis, and provides a new target for targeted therapy of breast cancer.

### Supplementary information

Supplementary Materia

Original western blots

## Data Availability

The datasets used and analyzed during the current study are available from the corresponding author upon reasonable request.
