# Supplementary material for: LncRNA HAGLROS promotes breast cancer evolution through miR-135b-3p/COL10A1 axis and exosome-mediated macrophage M2 polarization
Source: Cell Death Dis. 2024 Aug 28;15(8):633. doi: 10.1038/s41419-024-07020-x (PMC11358487; doi:10.1038/s41419-024-07020-x)
Supplement: Supplementary file 1 — Supplementary Materia [file 41419_2024_7020_MOESM1_ESM.pdf]

Supplementary material

A

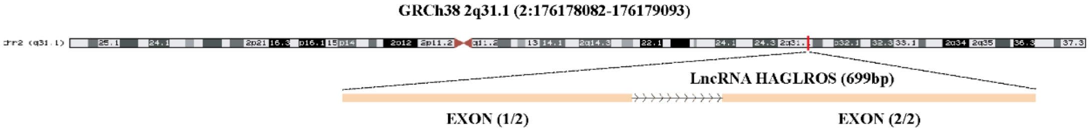

B

GCTCTCCGCGCCTCAGCGAGCGGCCAGGCTGCGGGCGGGGTGGGCTGGCGCTCCTGATCCACCTGGAACACTTTTGCAAAGACAGAC
GCGGTTCCTCCTACTTCAGAGCAAGTCTGGGCCAGCTGGGATCCGACCAGAAATCGCAAGCGGAGGAGACGCAGTAGCGCAGGCTGAG
CGCTAACTGAAGGCGCGACCTGAGCCCGGCGCCTGCTGGGGAGCTGCGCAGCCAGGACAGCGGTCGGCAGCACAGGGCCTGGGCGCA
GGGCCCGCCGTCACCACCTCACGTCGGAAGCCAGCACTGCTGCCCGCCAGCCCTGCCGCCTGCCCTCGGACTTCCCAGGGCGCCCAGG
GTCCTCCCAACGCGCCTGCACCCACACCCGCCCCTGAGCCACAGTGACCTTGCATTCCCCGCCCCCTGCCACACCTCCTGCGTGCCCGA
GTGTCACCCTTAAATACCGCTCTACACCCAGAGAGGGACGAATACACCTCTGAAGACAGGGCAAGACTAATTTCAATGGGTCAGAAGGTT
CTTGCAGGCACTGTTTTATTTAAGAGTGCCTATTTACTGGCAGGAGTATTTGTGTGGGAGGAAGTAGGCTAAGACTGCTGTTGAACTTGAA
GCTAAACTTGAATGCCATTTAAAATAACAATAAAAATATACTTAAAACACCAAAAAAAAAAAAAAAAAAAAAAAAAAAAA

C

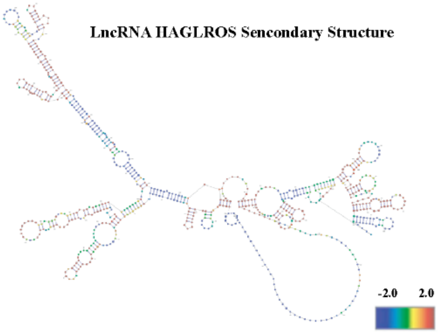

D

>ORF1

MGQKVLAGTVLFKSAYLLAGVFVWEEVG

>ORF2

MQGHCGSGAGVGAGALGGPWAPWEVRG

QAARPCAADRCPGCAAPQQAPGSGRAFS

>ORF3

MAFKFSFKFNSSLSLLPPTQILLPVNRHS

E

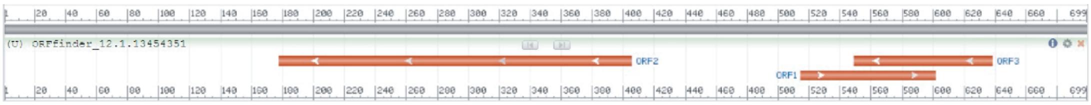

F

Protein coding potential

Metric	Raw result	Interpretation
PRIDE reprocessing 2.0	0	non-coding
Lee translation initiation sites	0	non-coding
PhyloCSF score	-415.7932	non-coding
CPAT coding probability	0.92%	non-coding
Bazzini small ORFs	0	non-coding

Supplementary Fig.1 The sequence, secondary structure and coding capacity of lncRNA HAGLROS. (A) Schematic diagram of the human lncRNA HAGLROS genomic locus. Orange rectangles represent exons. (B) The sequence of lncRNA HAGLROS. (C) The secondary structure of lncRNA HAGLROS from AnnoLnc database. (D) ORF Finder predicts ORFs of lncRNA HAGLROS. (E) The amino acid sequences of proteins. (F) The coding potential of lncRNA HAGLROS was measured by 5 different metrics.

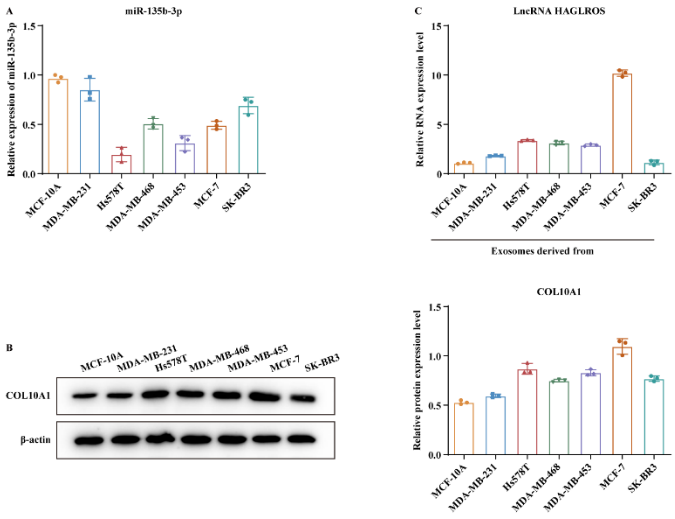

Supplementary Fig.2 Expression of miR-135b-3, COL10A1 and lncRNA HAGLROS in different cell lines and exosomes. (A) The expression of miR-135b-3p in breast epithelial cells and breast cancer cells was detected by qRT-PCR assay. GAPDH was used as an internal control. (B) The protein expression of COL10A1 in breast epithelial cells and breast cancer cells was detected by western blotting assay. (C) The expression of lncRNA HAGLROS in exosomes derived from breast epithelial cells and breast cancer cells was detected by qRT-PCR assay. GAPDH was used as an internal control.

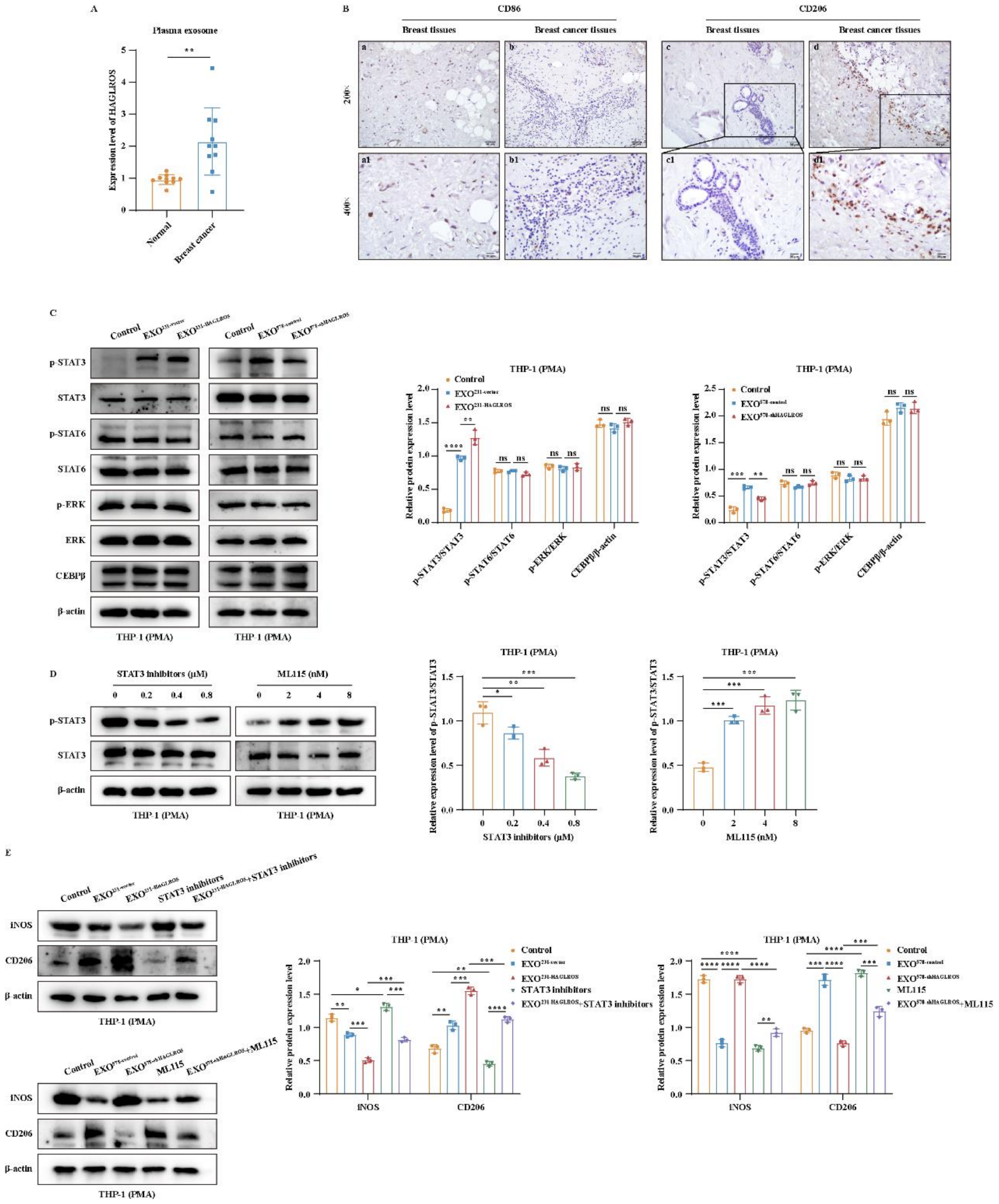

Supplementary Fig.3 Breast cancer cell-derived exosomal lncRNA HAGLROS promotes TAM/M2 polarization through the p-STAT3 signaling pathway. (A) The expression of lncRNA HAGLROS in plasma exosomes from breast cancer and healthy populations was examined by qRT-PCR assay. (B) The expression of CD86 and CD206 in breast cancer and paracancerous tissues was examined by IHC assay. (C) The protein expression levels of p-STAT3, STAT3, p-STAT6, STAT6, p-ERK, ERK, and CEBPβ in THP-1 (PMA) cells treated with exosomal lncRNA HAGLROS derived from breast cancer cells were determined by western blotting assay. (D) The protein expression

levels of p-STAT3 and STAT3 in THP-1 (PMA) cells treated with different concentrations of STAT3 inhibitor or agonist (ML115) were determined by western blotting assay. (E) Macrophages were cultured with exosomes derived from breast cancer cells differentially expressing lncRNA HAGLROS and STAT3 inhibitors/agonists, and the expression of TAM/M1 and TAM/M2 related markers in macrophages was detected by western blotting assay.

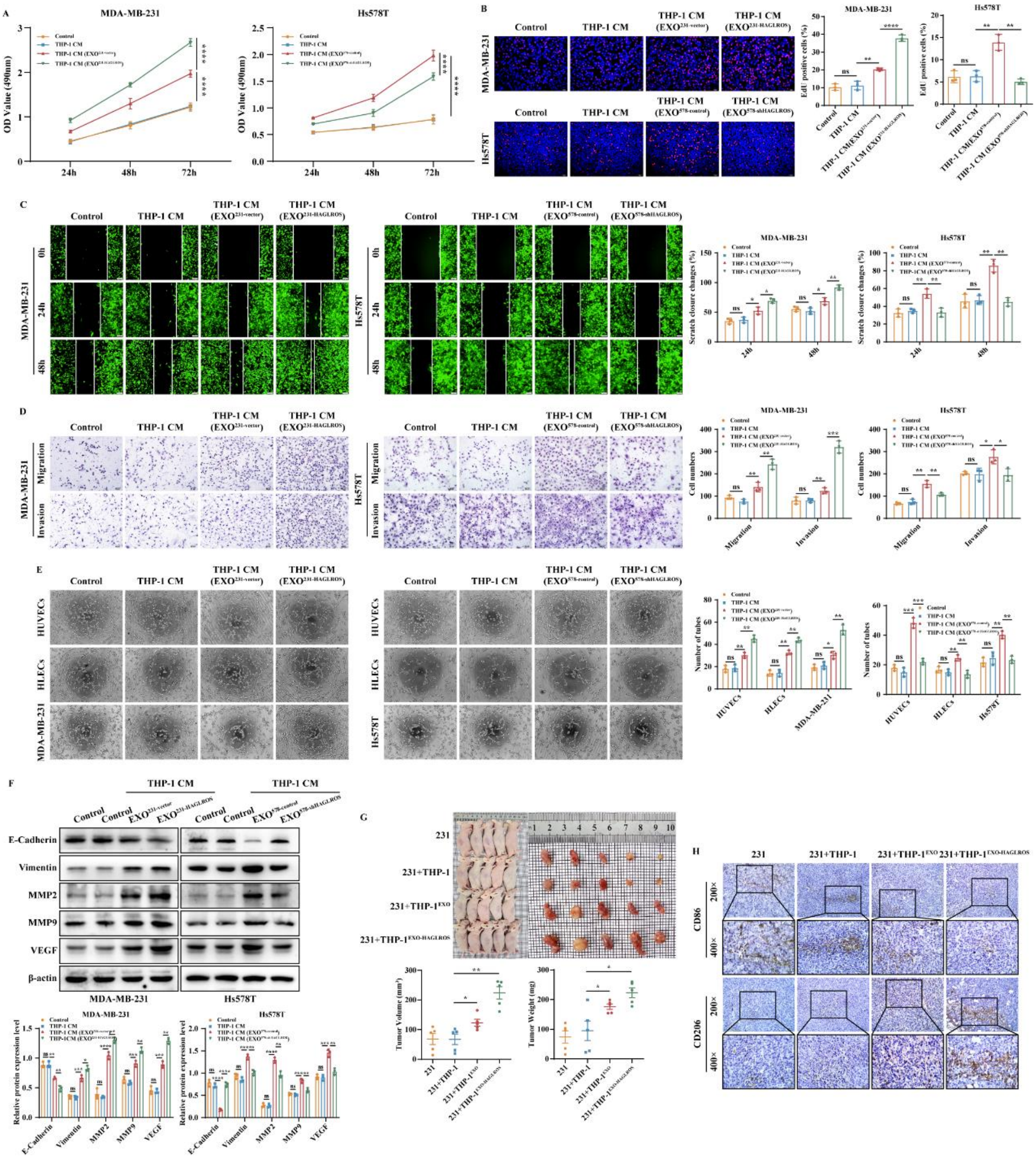

Supplementary Fig.4 Exosomal lncRNA HAGLROS promotes the malignant progression of breast cancer by inducing TAM/M2 polarization. (A-B) The effect of macrophage supernatants treated with exosomes differentially expressing lncRNA

HAGLROS on the proliferation ability of breast cancer cells was detected by MTT and EdU assays. (C-D) The effect of macrophage supernatants treated with exosomes differentially expressing lncRNA HAGLROS on the migration and invasion ability of breast cancer cells was detected by wound healing and Transwell assays. (E) The effect of macrophage supernatants treated with exosomes differentially expressing lncRNA HAGLROS on the angiogenesis ability of HUVECs, HLECs and breast cancer cells was detected by endothelial tube formation and VM assays. (F) The effect of macrophage supernatants treated with exosomes differentially expressing lncRNA HAGLROS on the expression levels of EMT and angiogenesis-related markers in breast cancer cells was detected by western blotting assay. β-actin was used as an internal control. (G) Representative images of xenograft tumors in nude mice co-injected with breast cancer cells and exosome-treated macrophages with different lncRNA HAGLROS levels, as well as the volume and weight of the tumors (n=5 per group). (H) The expression of CD86 and CD206 in the xenograft tumor tissues was detected by IHC staining.

Supplementary Table 1. The sequences of siRNAs.

**Sequence Name**	**Sequence（5’ to 3’）**
si-NC	UUCUCCGAACGUGUCACGU
COL10A1 (siRNA#1)	GCAACAGCAUUAUGACCCA
COL10A1 (siRNA#2)	CCAAGACACAGUUCUUCAU
COL10A1 (siRNA#3)	CCUACACCAUAAAGAGUAA

Supplementary Table 2. qRT-PCR primer sequences.

**Primer name**	**Primer sequences（5’ to 3’）**
LncRNA HAGLROS-F	CTCCTGATCCACCTGGAACAC
LncRNA HAGLROS-R	CTCCGCTTGCGATTTCTGG
LncRNA GAPDH-F	GTGCTCAACCAGTTGGCACC
LncRNA GAPDH-R	AGCCTCGCTCCACCTGACTT

hsa-miR-135b-3p-F	ATGTAGGGCTAAAAGCCATGGG
COL10A1-F	ATGCTGCCACAAATACCCTTT
COL10A1-R	GGTAGTGGGCCTTTTATGCCT
ULBP1-F	TAAGTCCAGACCTGAACCACA
ULBP1-R	TCCACCACGTCTCTTAGTGTT
SIX4-F	AGCAGCTCTGGTACAAGGC
SIX4-R	CTTGAAACAATACACCGTCTCCT
SHISA9-F	TCGGGCGACTTCATCTTCTG
SHISA9-R	TGATGTAGACGATCAGGTTGGT
CKAP2L-F	GAGCCAAAACACCAAGCCTTA
CKAP2L-R	GGAGTTTAATGCTGATGGACCTT
DYNAP-F	CCAGCCGCCAATCACACAT
DYNAP-R	TGGGAGAGACATCACTGGTTAT
GAPDH-F	GGAGCGAGATCCCTCCAAAAT
GAPDH-R	GGCTGTTGTCATACTTCTCATGG
CD206-F	TCCGGGTGCTGTTCTCCTA
CD206-R	CCAGTCTGTTTTTGATGGCACT
CD163-F	TTTGTCAACTTGAGTCCCTTCAC
CD163-R	TCCCGCTACACTTGTTTTCAC
Arg-1-F	GTGGAAACTTGCATGGACAAC
Arg-1-R	AATCCTGGCACATCGGGAATC
CD86-F	CTGCTCATCTATACACGGTTACC
CD86-R	GGAAACGTCGTACAGTTCTGTG
iNOS-F	TTCAGTATCACAACCTCAGCAAG
iNOS-R	TGGACCTGCAAGTTAAAATCCC

Supplementary Table 3. Antibody used in this work.

**Antibody name**	**Catalog number**	**Manufacturer**	**Origin**
E-Cadherin	14472	Cell Signaling Technology	USA

ZO-1	8193	Cell Signaling Technology	USA
Vimentin	6260	Santa Cruz	USA
Snail	3879	Cell Signaling Technology	USA
Slug	166476	Santa Cruz	USA
Twist	81417	Santa Cruz	USA
MMP2	13594	Santa Cruz	USA
MMP9	393859	Santa Cruz	USA
VEGF	7269	Santa Cruz	USA
β-actin	CW0096	CWBIO	China
COL10A1	ab182563	Abcam	USA
IgG	30000-0-AP	Proteintech	USA
Ki67	23900	Santa Cruz	USA
TSG101	79642	Santa Cruz	USA
CD9	13118	Santa Cruz	USA
iNOS	13120	Cell Signaling Technology	USA
CD86	28347	Santa Cruz	USA
CD206	24595	Cell Signaling Technology	USA
APC-CD68	333809	Biolegend	USA
FITC-CD86	374203	Biolegend	USA
PE-CD206	321105	Biolegend	USA
STAT3	9139	Cell Signaling Technology	USA
p-STAT3	9145	Cell Signaling Technology	USA
STAT6	374021	Santa Cruz	USA
p-STAT6	56554	Cell Signaling Technology	USA
ERK	48303	Cell Signaling Technology	USA
p-ERK	4695	Cell Signaling Technology	USA
CEBPβ	7962	Santa Cruz	USA
Goat anti-mouse	2305	ZSGB-BIO	China

Goat anti-rabbit	2301	ZSGB-BIO	China

Supplementary Table 4. LncRNA HAGLROS expression in breast cancer patients.

Diagnosis	No. of cases	LncRNA HAGLROS expression				Positive rates (+to++)	Strongly positive rates (+++)
		–	+	++	+++		
Breast cancer tissues	99	17	28	18	36	82.8%***	36.4%***
Breast tissues	79	58	9	10	2	26.6%	2.5%

Supplementary Table 5. Correlation between lncRNA HAGLROS expression and the clinicopathological features of breast cancer patients.

Variables	No.of cases	LncRNA HAGLROS Strongly positive cases (%)	χ^2	*p* value
Age(years)			2.347	0.126
<50	23	5 (21.7)		
≥50	79	29 (39.2)		
Histological grade			2.834	0.092
Grade 1	-	-		
Grade 2	63	19 (30.2)		
Grade 3	29	14 (48.3)		
Tumor size			0.000	1.000
T1-T2	87	29 (33.3)		
T3-T4	3	1 (33.3)		
LN metastasis			5.366	0.021*
Negative	48	10 (20.8)		
Positive	36	16 (44.4)		
Metastasis			9.971	0.002**
Negative	83	26 (31.3)		
Positive	13	10 (76.9)		

TNM stage			30.810	0.000***
I-II	59	9 (15.3)		
III-IV	31	23 (74.2)		
ER			4.295	0.038*
Negative	21	2 (9.5)		
Positive	48	16 (33.3)		
PR			3.404	0.065
Negative	28	4 (14.3)		
Positive	41	14 (34.1)		
HER2			0.081	0.776
Negative	21	5 (23.8)		
Positive	48	13 (27.1)		
Ki67			1.573	0.210
Negative	50	11 (22.0)		
Positive	19	7 (36.8)		

Supplementary Table 6. Cox regression model analysis of the clinicopathological features in breast cancer patients.

Factors	B	SE	Wald	HR	95%CI		*p* value
					Lower	Upper	
**Univariate survival analyses**							
Age	3.354	3.570	0.883	28.615	0.026	31272.955	0.347
Histological grade	0.591	0.825	0.513	1.805	0.359	9.092	0.474
Tumor size	-3.115	6.008	0.269	0.044	0.000	5767.116	0.604
LN metastasis	0.993	0.731	1.846	2.700	0.644	11.318	0.174
Metastasis	1.978	0.675	8.571	7.225	1.922	27.154	0.003**
TNM stage	3.111	1.071	8.435	22.448	2.750	183.232	0.004**
ER	-0.509	0.673	0.572	0.601	0.161	2.248	0.449
PR	-0.381	0.675	0.319	0.683	0.182	2.565	0.572

HER2	0.219	0.803	0.074	1.245	0.258	6.007	0.785
KI67	-0.371	0.802	0.214	0.690	0.143	3.323	0.644
LncRNA HAGLROS expression	2.485	0.805	9.533	11.995	2.478	58.068	0.002**
**Multivariate survival analyses**							
TNM stage	1.413	1.338	1.114	4.107	0.298	56.586	0.291
Metastasis	0.376	0.810	0.215	1.456	0.298	7.117	0.643
LncRNA HAGLROS expression	2.506	1.268	3.910	12.261	1.022	147.040	0.048*

63
