# Supplementary material for: LncRNA HAGLROS promotes breast cancer evolution through miR-135b-3p/COL10A1 axis and exosome-mediated macrophage M2 polarization
Source: Cell Death Dis. 2024 Aug 28;15(8):633. doi: 10.1038/s41419-024-07020-x (PMC11358487; doi:10.1038/s41419-024-07020-x)

Figure 3A

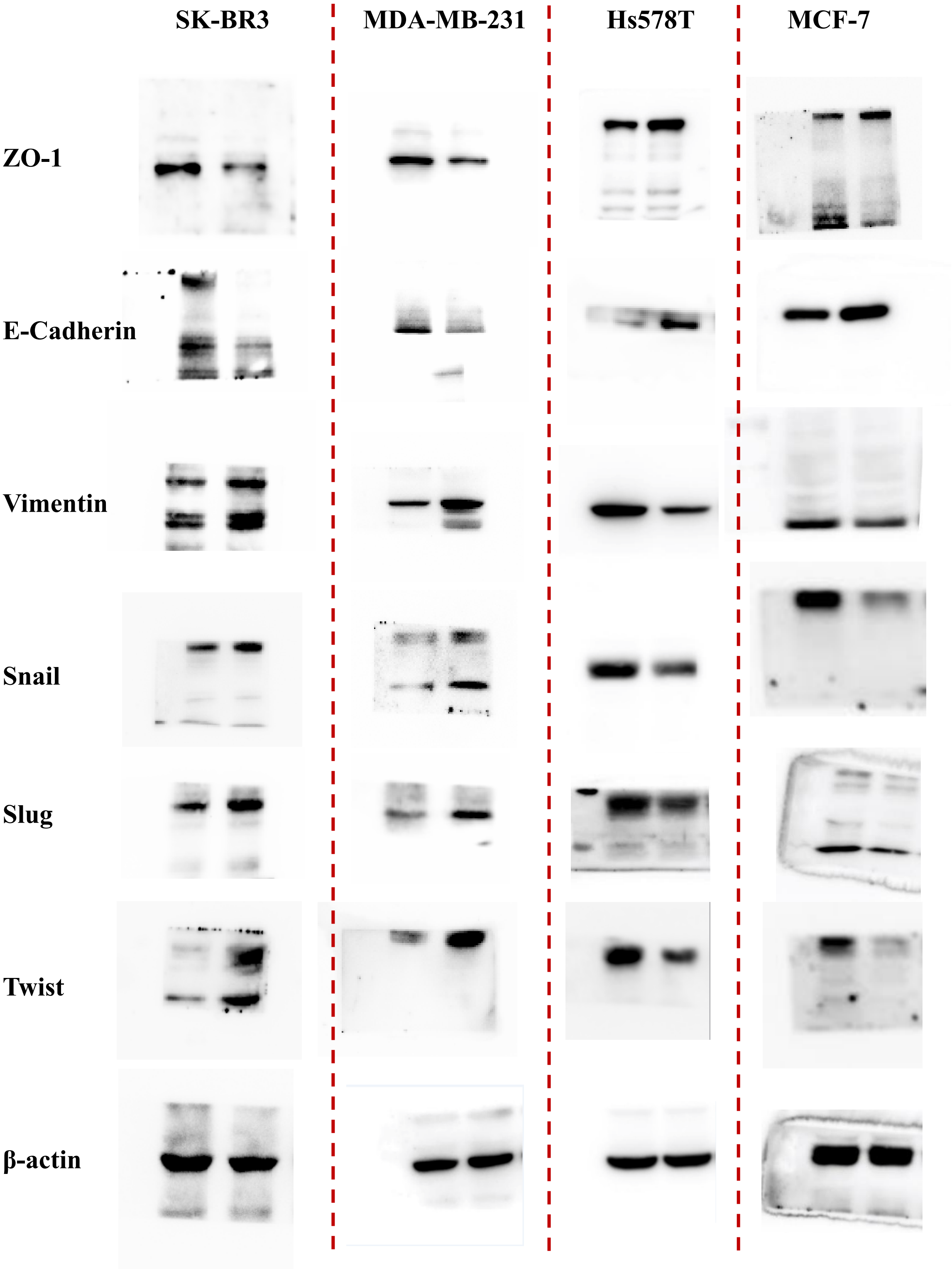

SK-BR3
MDA-MB-231
Hs578T
MCF-7
ZO-1
E-Cadherin
Vimentin
Snail
Slug
Twist
β-actin

Figure 3D

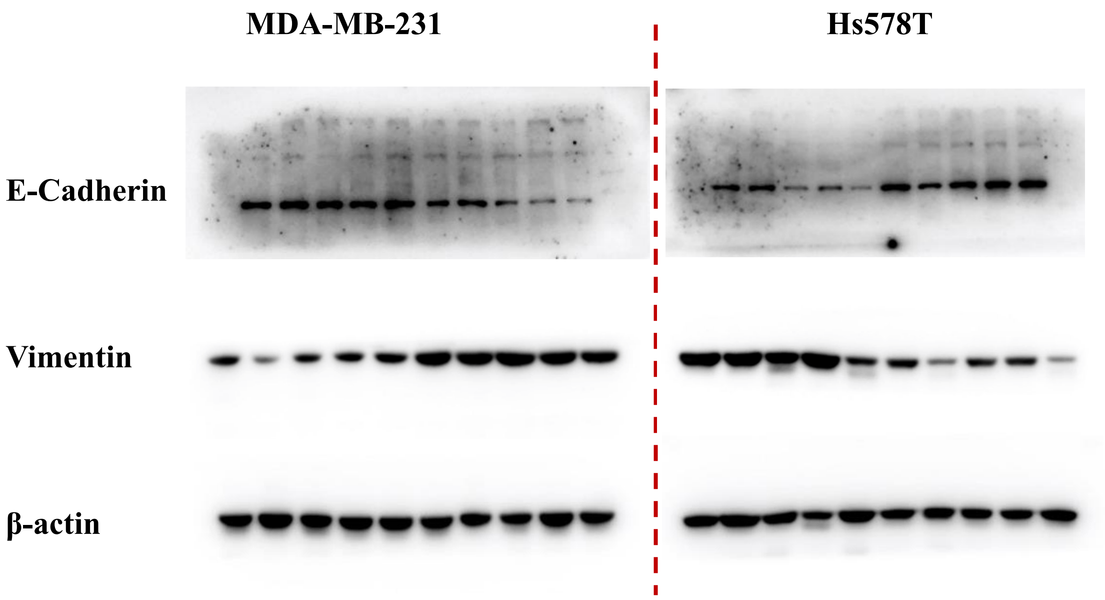

Figure 3H

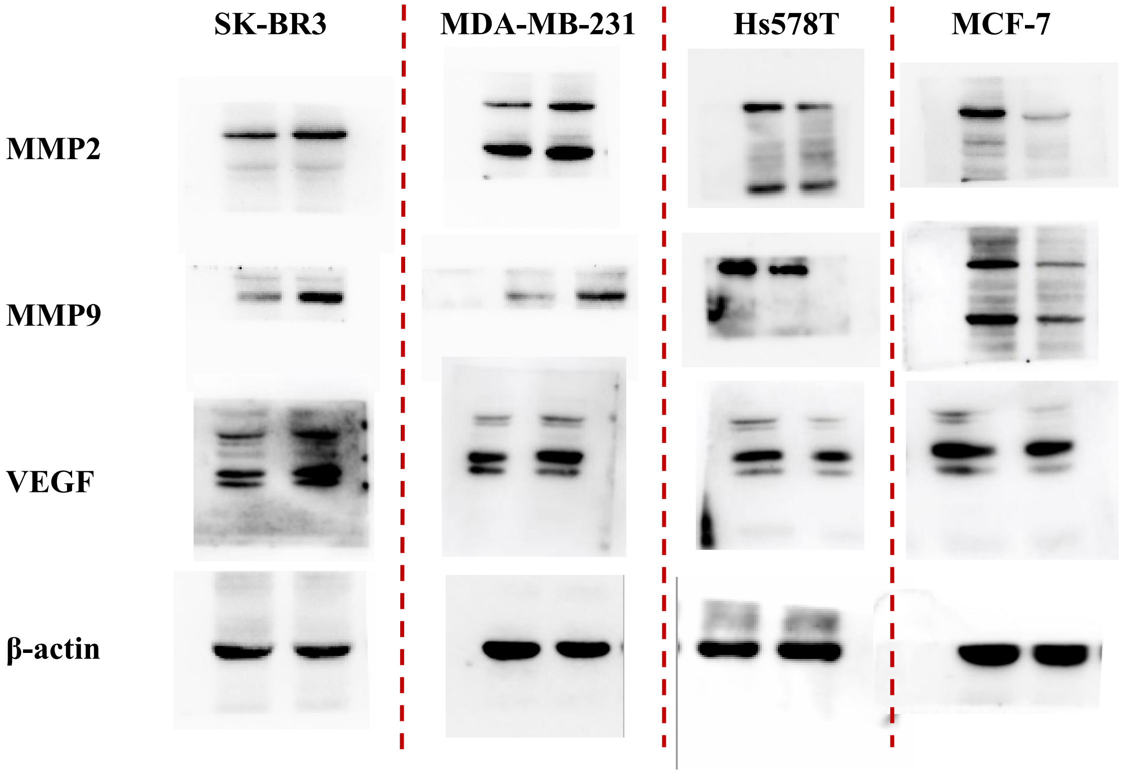

Figure 5F

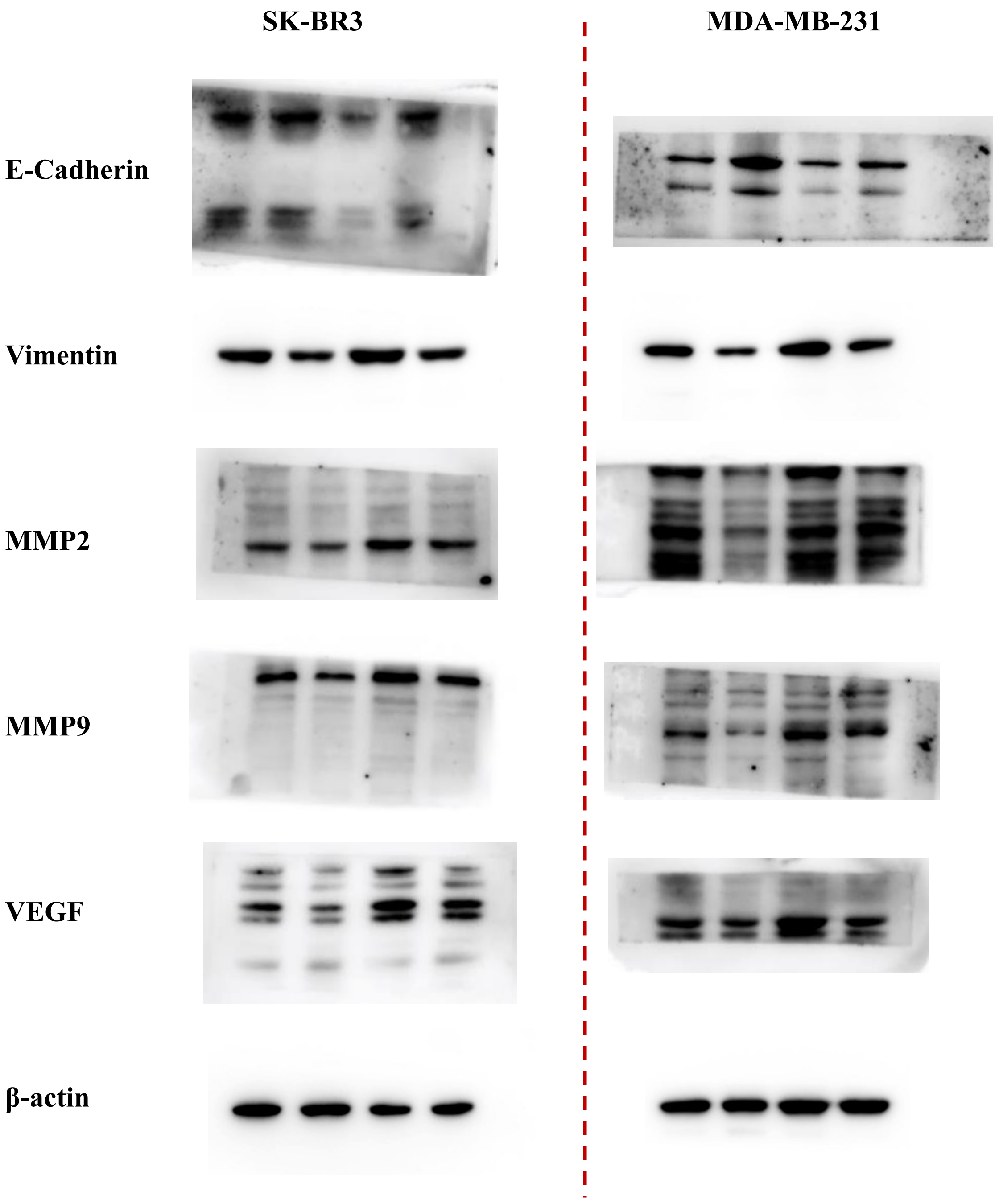

Figure 5F

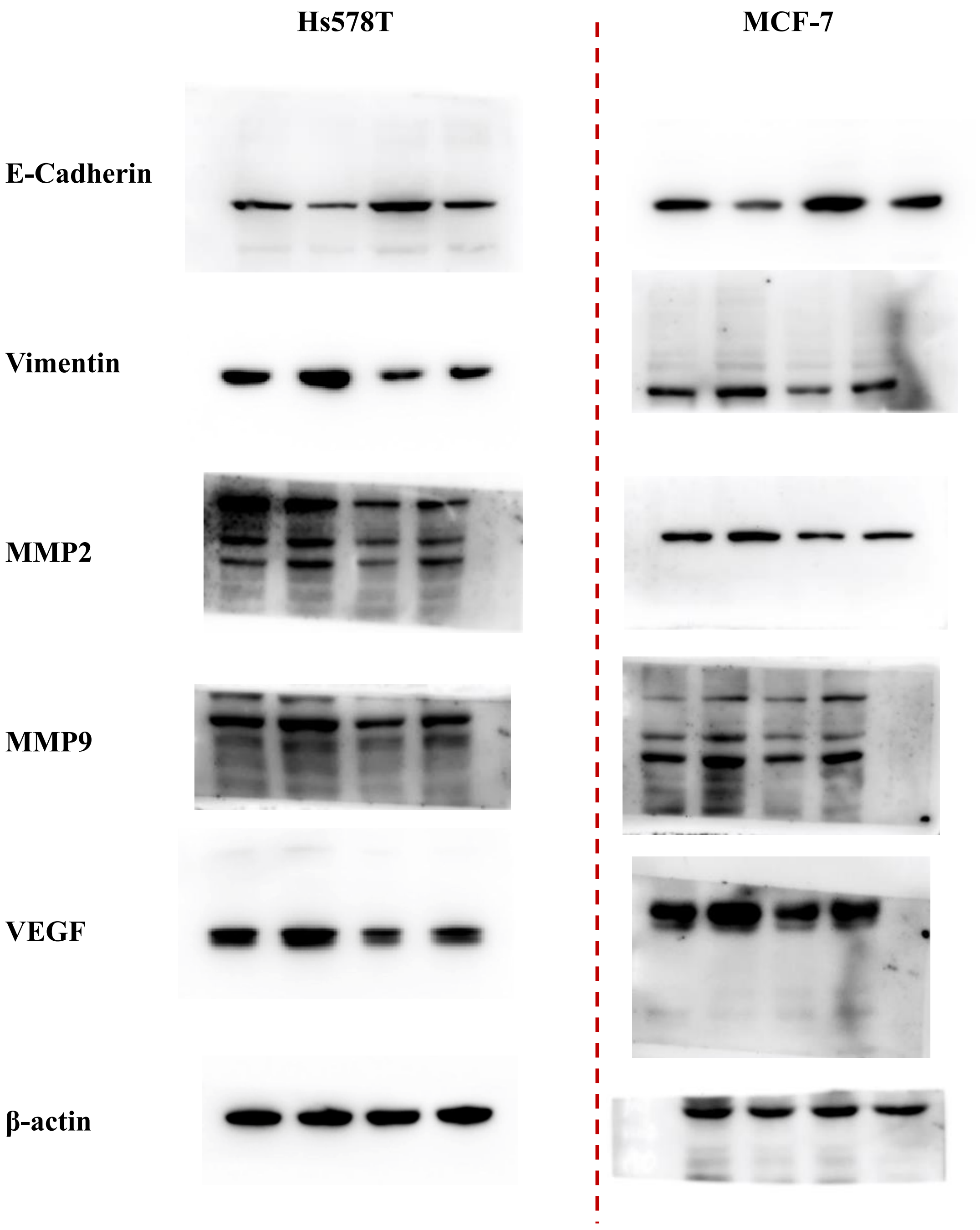

Figure 6F

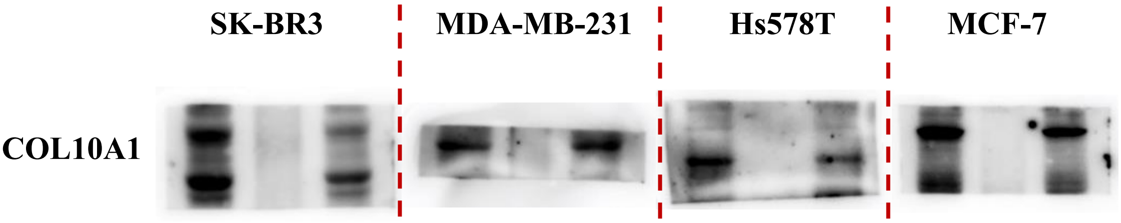

Figure 6G

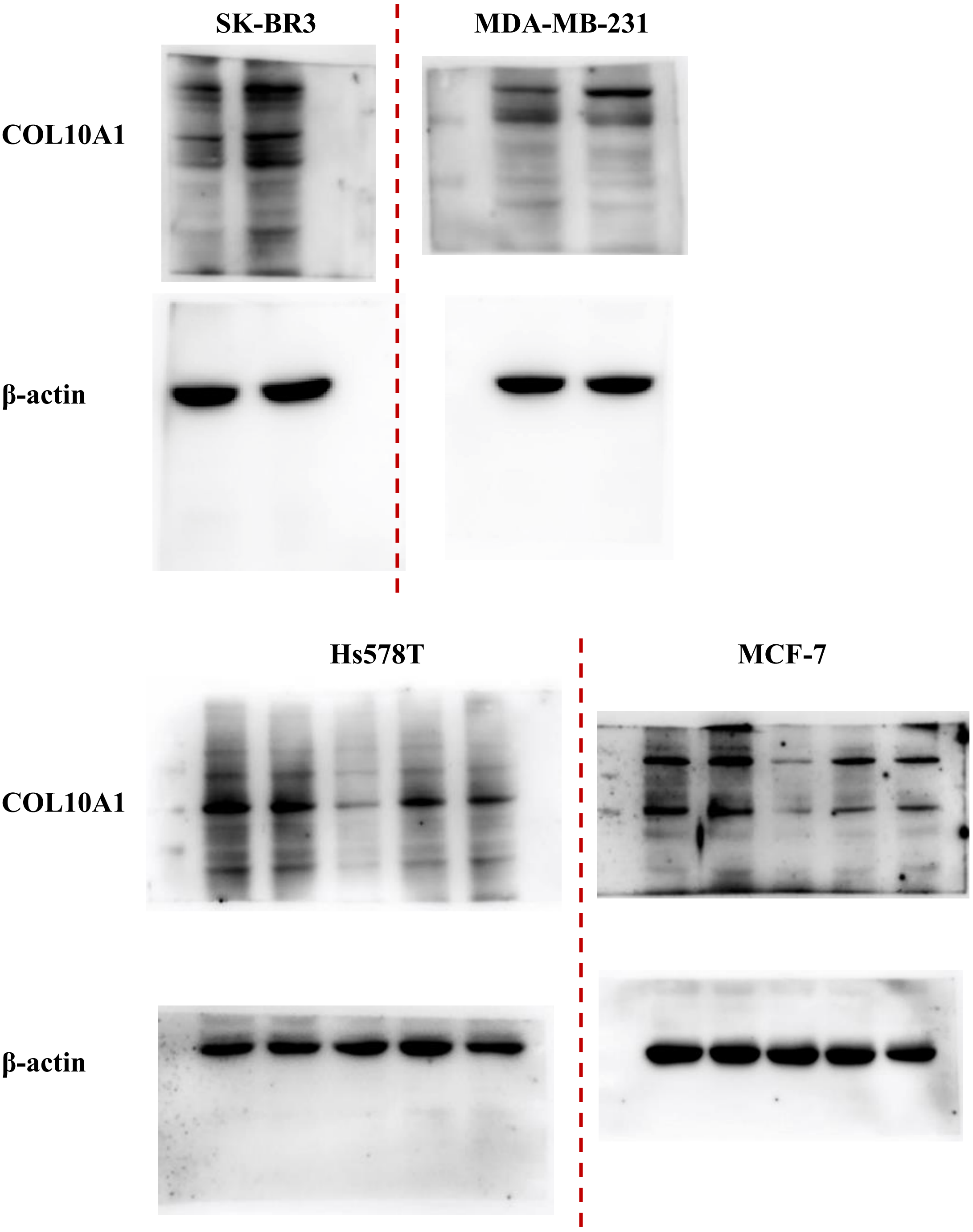

Figure 7F

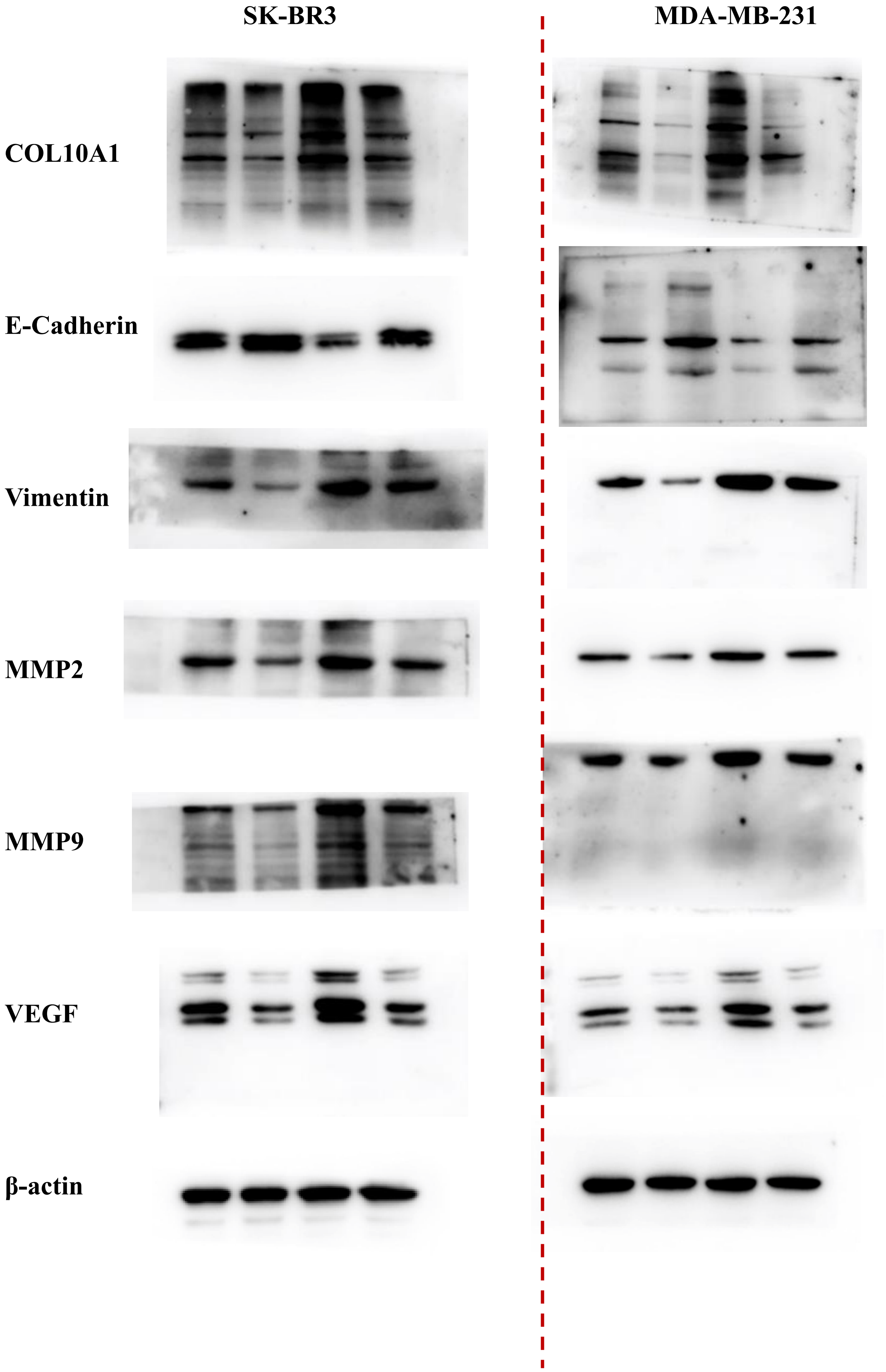

SK-BR3
MDA-MB-231
COL10A1
E-Cadherin
Vimentin
MMP2
MMP9
VEGF
β-actin

Figure 7F

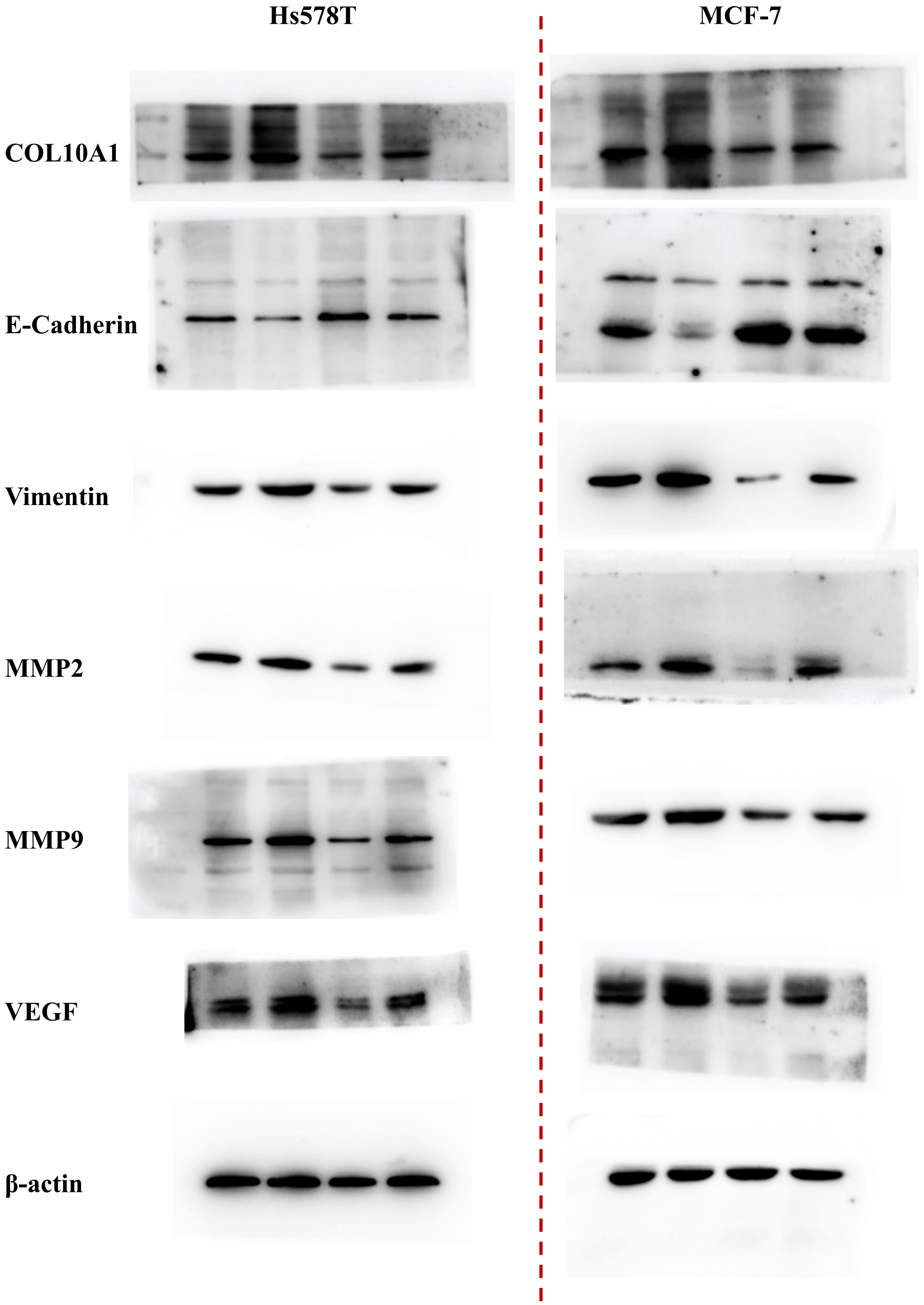
Hs578T
MCF-7
COL10A1
E-Cadherin
Vimentin
MMP2
MMP9
VEGF
β-actin

Figure 7H

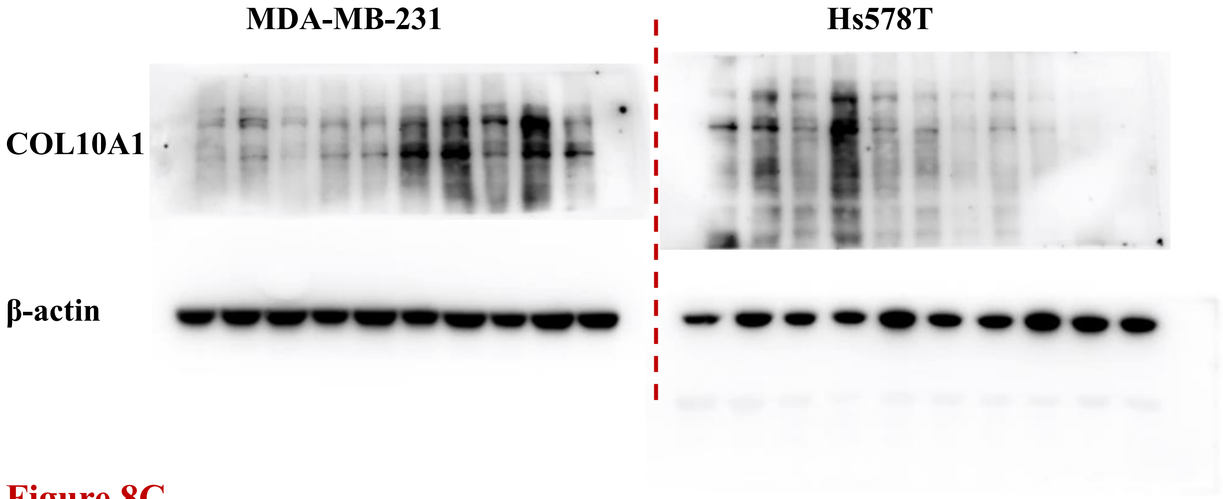

Figure 8C

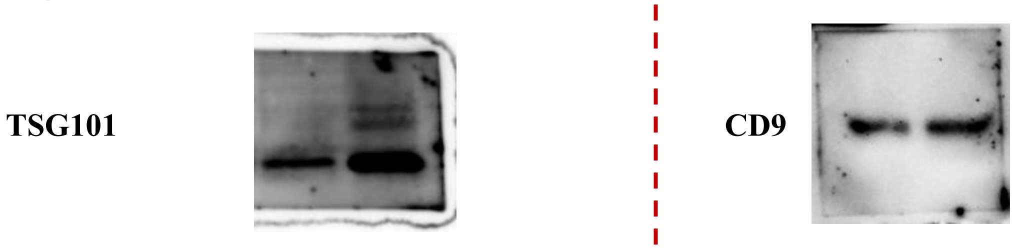

Figure 8G

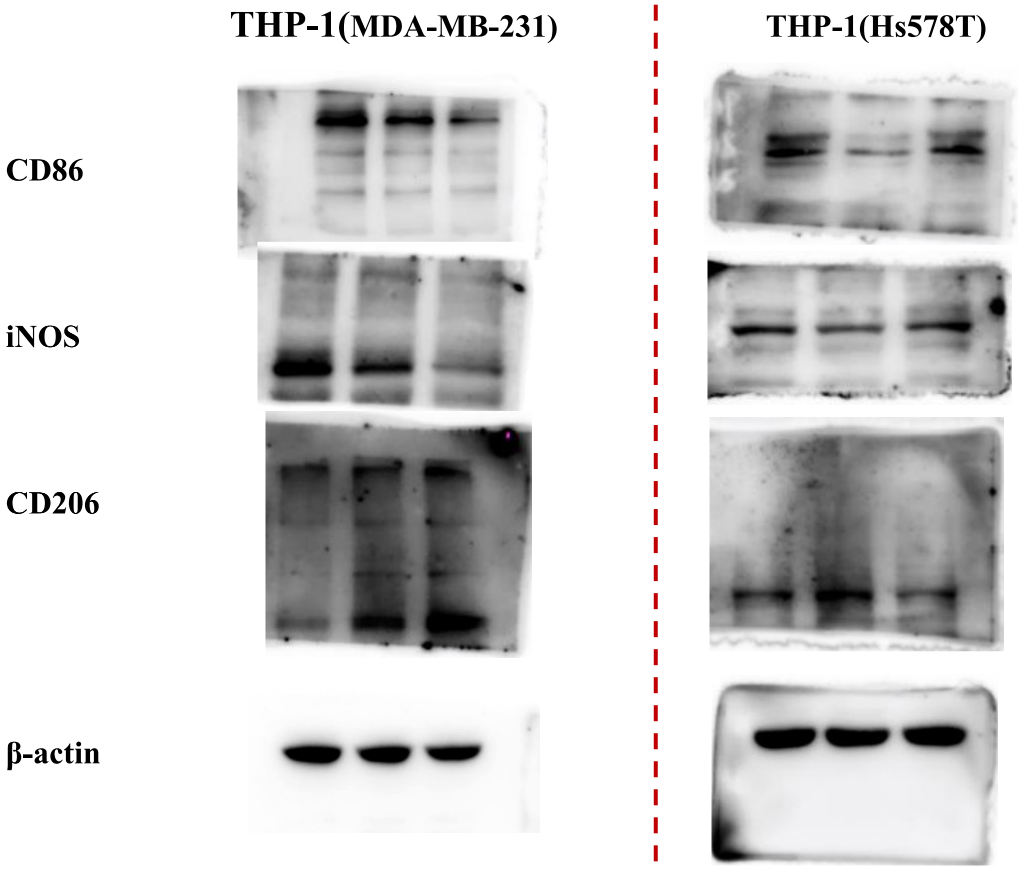

Supplementary Figure 2A

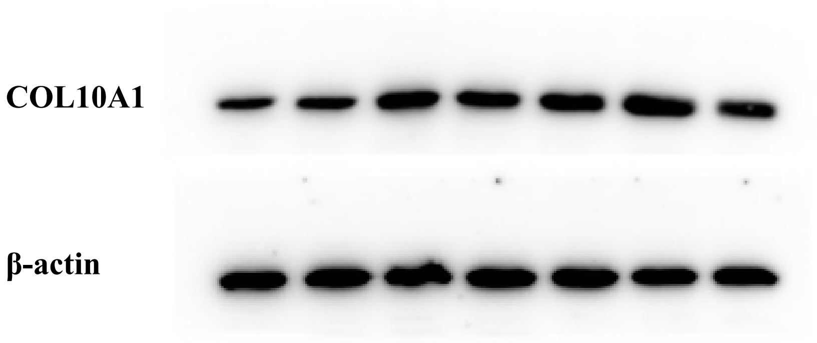

Supplementary Figure 3C

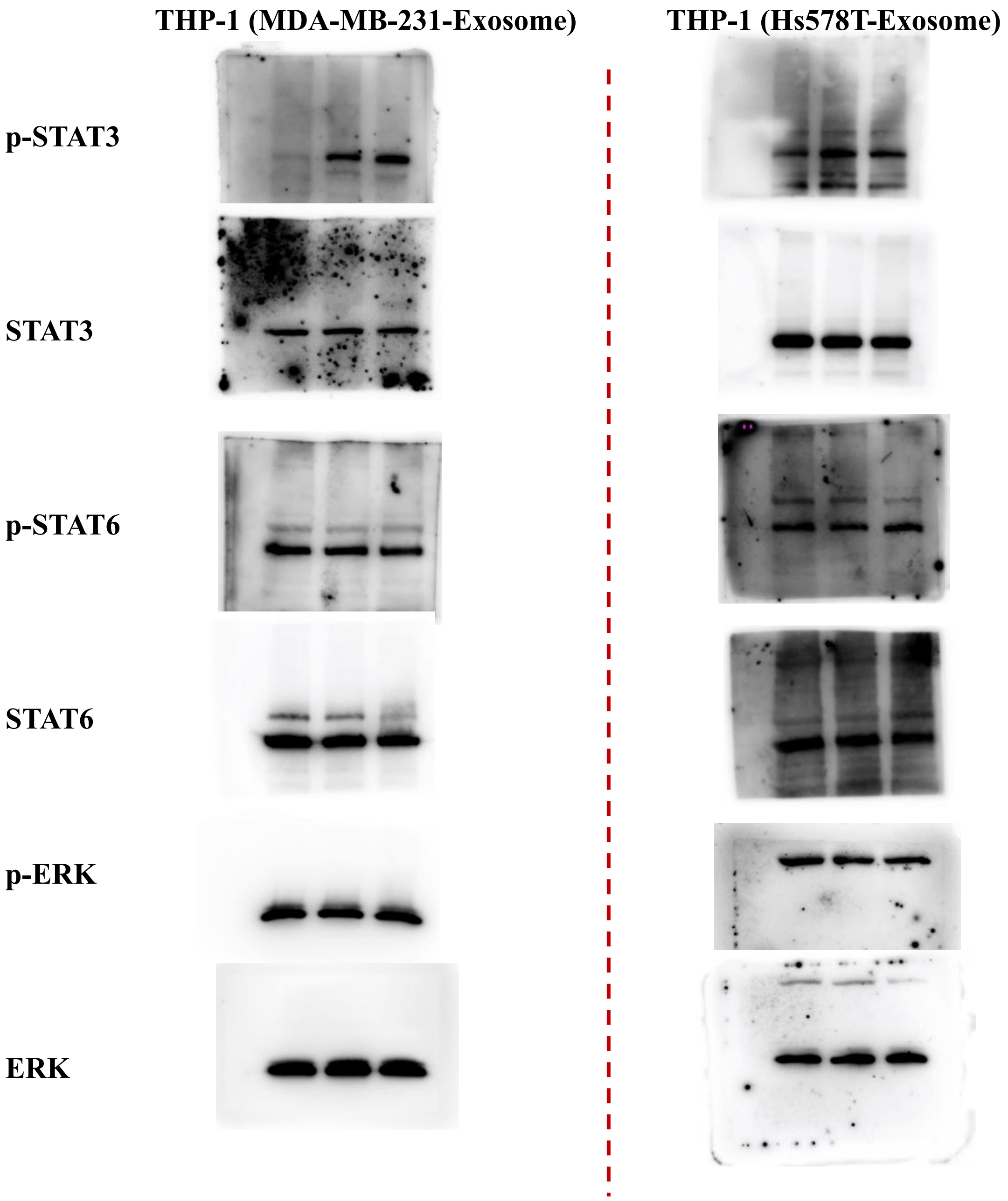

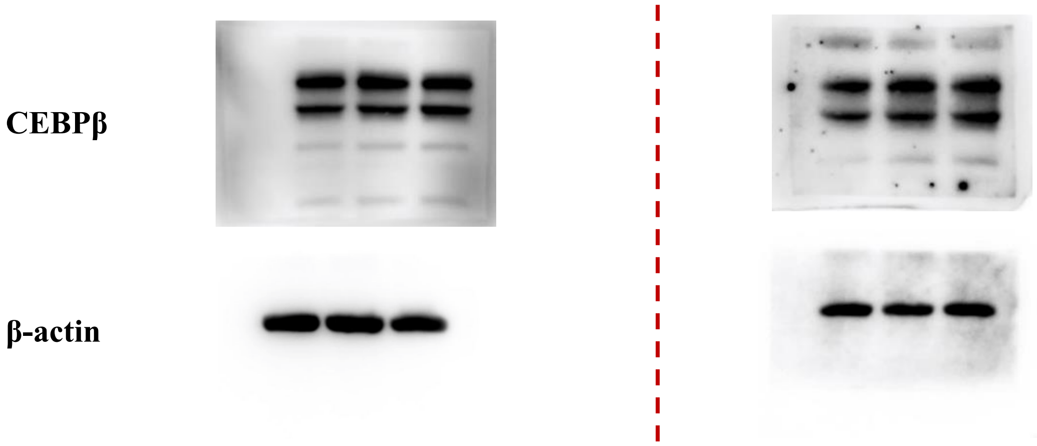

Supplementary Figure 3D

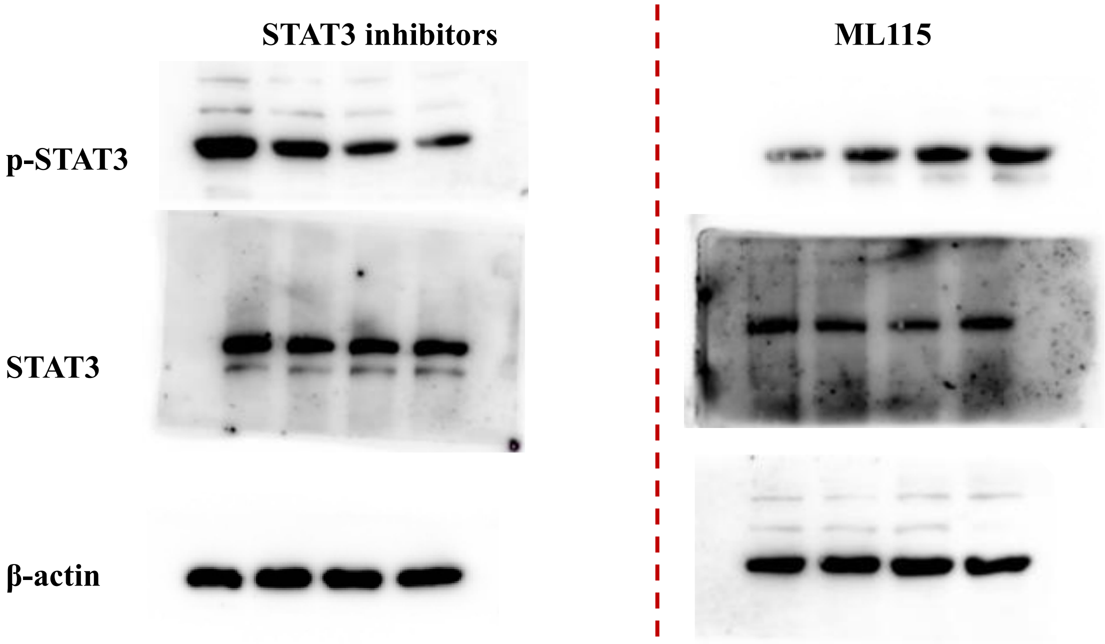

Supplementary Figure 3E

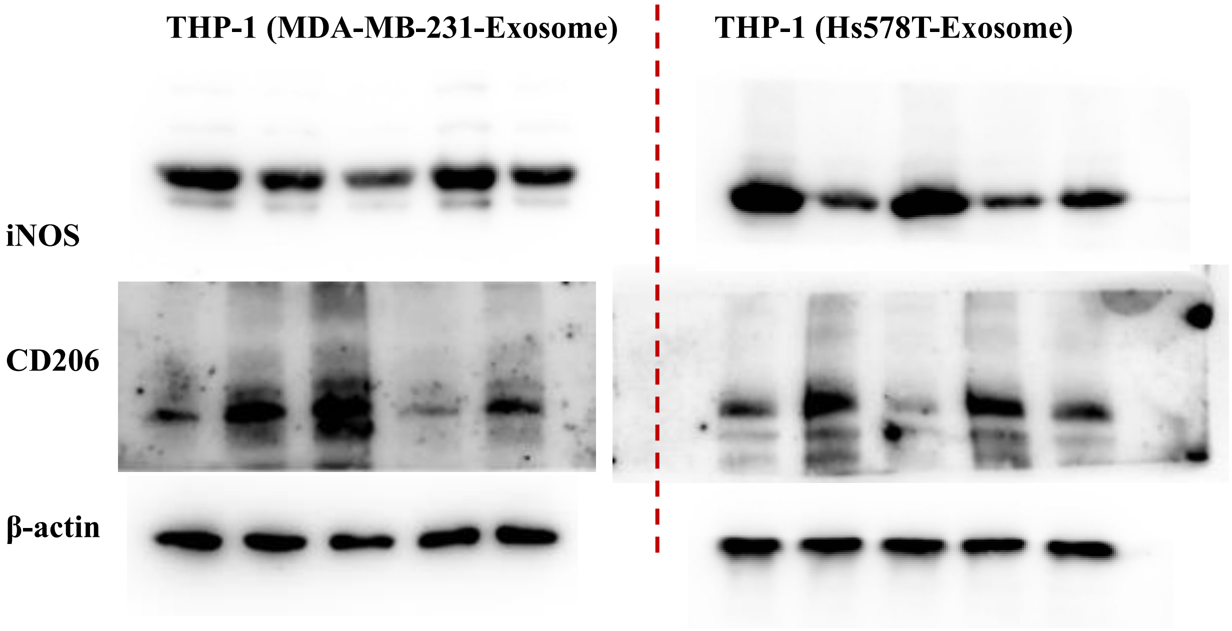

Supplementary Figure 4F

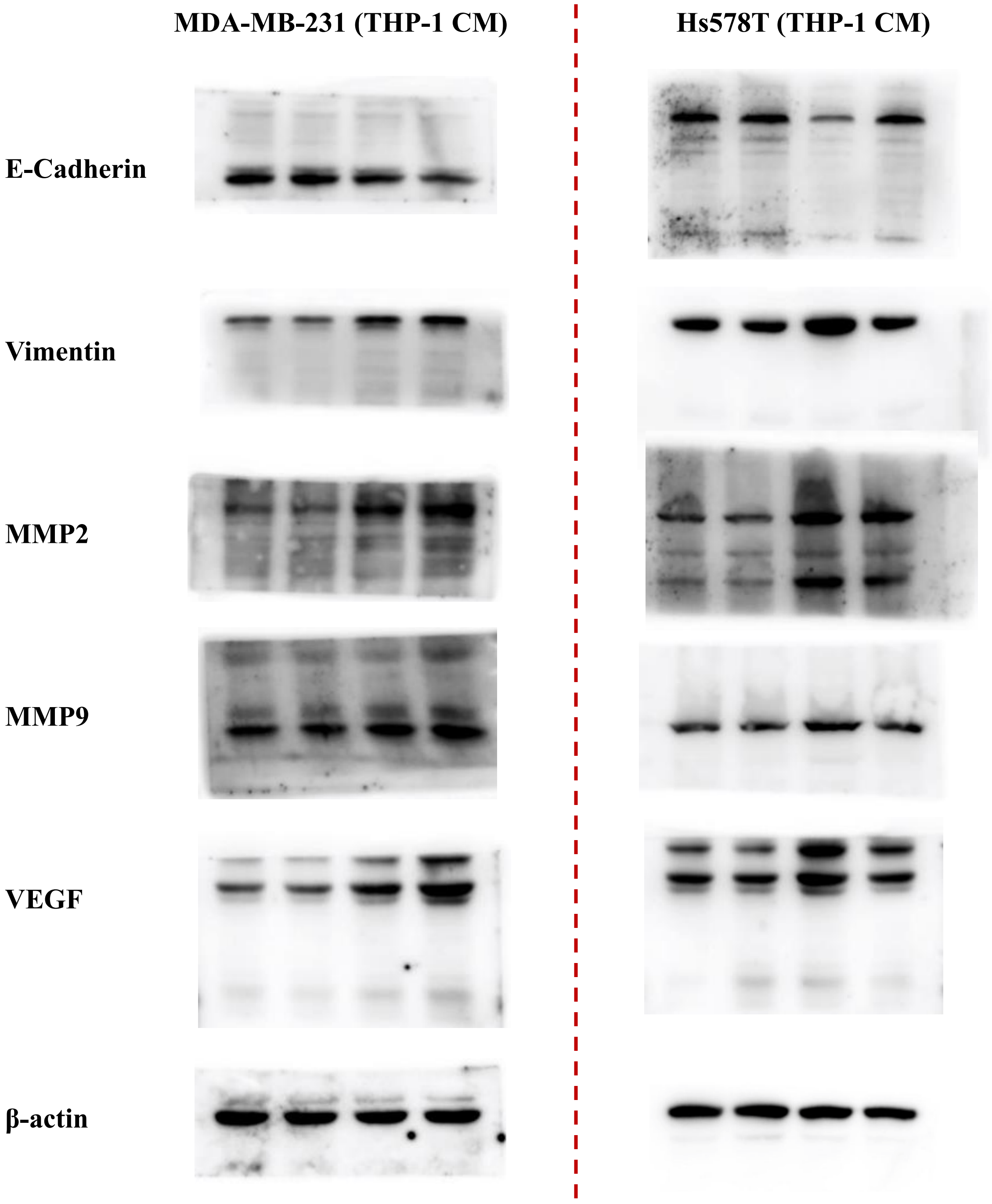

Supplement: Supplementary file 2 — Original western blots [file 41419_2024_7020_MOESM2_ESM.pdf]
